# H2B gene family: A prognostic biomarker and correlates with immune infiltration in glioma

**DOI:** 10.3389/fonc.2022.966817

**Published:** 2022-10-25

**Authors:** Jingnan Jia, Zhaocheng Han, Xueke Wang, Xiancheng Zheng, Shurui Wang, Yinglin Cui

**Affiliations:** ^1^ The Second Clinical Medical School, Henan University of Chinese Medicine, Zhengzhou, China; ^2^ Department of Chinese Medicine, JiRen Hospital of Chinese Medicine, Zhengzhou, China; ^3^ FuXing Hospital, Capital Medical University, Beijing, China; ^4^ Department of Encephalopathy, Henan Province Hospital of Traditional Chinese Medicine, Zhengzhou, China

**Keywords:** H2B family genes, glioma, pan-cancer analysis, prognostic factors, immune infiltration

## Abstract

The current prognosis of glioma is unfavorable and effective treatments remain limited. However, bioinformatics has created new opportunities for improving glioma treatment. Research indicates that H2B is involved in the pathological process of cancer. Thus, this study conducted bioinformatic analyses of the H2B gene family to evaluate whether these genes can play a role in predicting prognosis and are associated with immune infiltration. High expression of H2B genes was observed in cholangiocarcinoma, esophageal carcinoma, glioblastoma multiforme (GBM), head and neck squamous cell carcinoma, and other cancers. In addition, a rise in H2B gene expression was correlated with an increase in glioma grade. In the Cancer Genome Atlas (TCGA), the Chinese Glioma Genome Atlas (CGGA) database and multiple datasets from the Gene Expression Omnibus (GEO), high expression of H2B gene family members predicted poor prognosis of a variety of tumors including glioma. In particular, high H2BC5, H2BC9, H2BC11, and H2BC21 expression was associated with poor glioma prognosis. H2BC9, H2BC11, and H2BC12 expression were also positively correlated with both immune and stromal scores. Enrichment analysis indicated that H2B family genes may be involved in the pathological process of glioma using various pathways including the cell cycle and immune response. H2B-specific siRNAs were used to verify the role of H2BC5, H2BC9, H2BC11, and H2BC21 expression on cell cycle distribution. In summary, H2BC5, H2BC9, H2BC11, and H2BC21 were independent prognostic indicators of glioma, and H2BC9 and H2BC11 may correlate with tumor immunity.

## Introduction

Glioma is one of the most invasive primary tumors in the central nervous system of adults. The average annual incidence of glioma is approximately five cases per 100,000 people, accounting for 24.5% of all brain tumors and 80.9% of malignant tumors (1). Glioma can cause patients to suffer from many neurological and functional limitations which greatly impact their quality of life (2), and also put a huge economic burden on social and medical resources (3). At the same time, clinicians have trouble encountering glioma because they are difficult to cure using conventional treatments. While several new treatment strategies, including recombinant poliovirus (4) and minimally invasive techniques (5), are being used to improve the prognosis of glioma patients, the overall survival (OS) time requires further improvement.

Molecular markers are shown to provide powerful prognostic information. Through the use of bioinformatics, the World Health Organization was able to include the mutation status of isocitrate dehydrogenase as a biomolecular marker for glioma diagnosis (6). More studies have adopted high throughput sequencing technologies, including microarrays, to analyze biomarkers and have made significant contributions to cancer pathology research. Thus, in order to develop effective diagnostic and treatment methods for glioma, more research is necessary at the molecular level.

**Figure 1:**
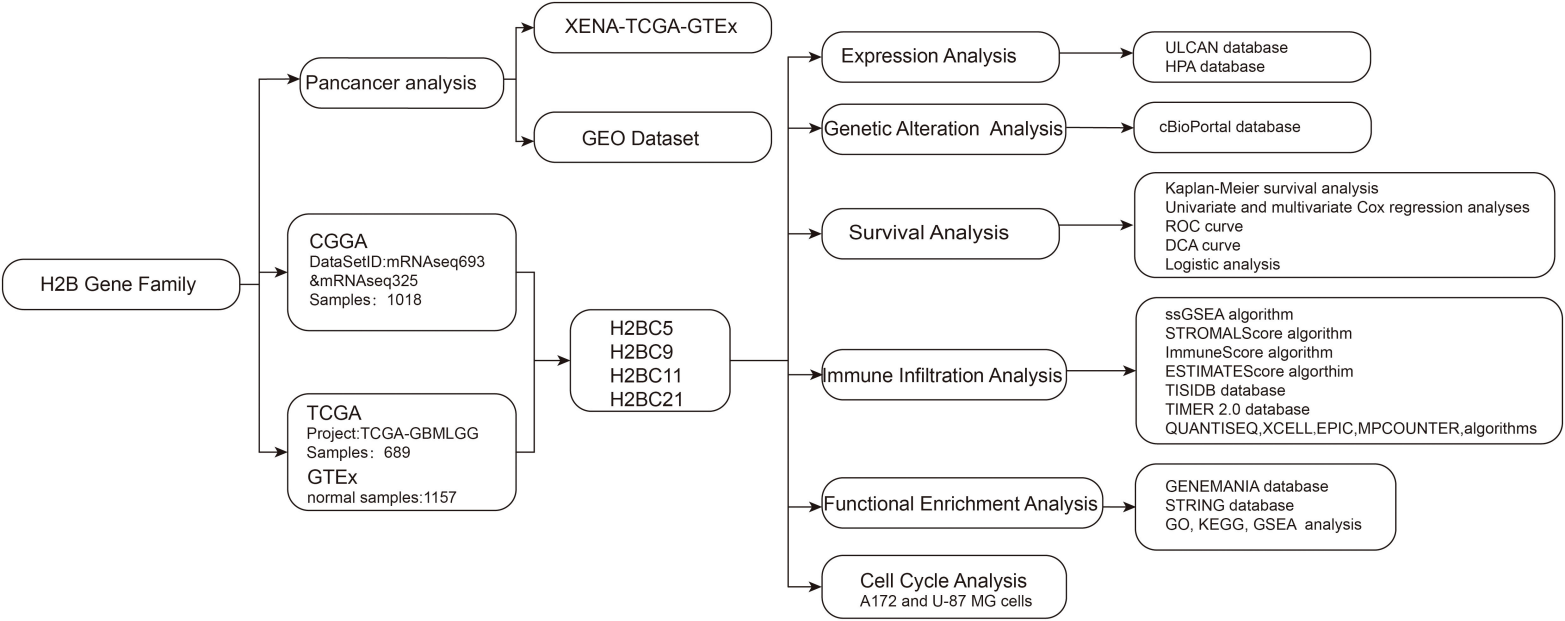
Flow chart.

This study explored differentially expressed GBM and low-grade glioma (LGG) genes using data obtained from public cancer databases to characterize and assess the effects of significant genes. Particular focus was placed on the H2B Clustered Histone gene family and its potential role in glioma prognosis and immune infiltration. Histone H3.3 gene mutation is correlated with the most significant pathogenesis of diffuse midline glioma, so it is important to better understand H2B gene family function as these are also histone-coding genes. Given that recent studies have defined an important role for H2BC12 in low-grade glioma (7), this study focuses on other members of the H2B gene family. The procedure of this study was presented in the flow chart (**Figure 1**).

## Methods

### Data sources and processing

The XENA-TCGA_GTEx (pan-cancer) cohorts were downloaded from UCSC XENA (https://xenabrowser.net/datapages/), and the TCGA_GTEx-GBMLGG (glioma) dataset was received from the National Cancer Institute’s Genomic Data Commons (https://portal.gdc.cancer.gov/). The corresponding normal tissue GTEx data were uniformly processed into RNAseq data in the transcripts per million reads (TPM) format using the Toil process (8). MRNAseq_693 and the mRNAseq_325 datasets were downloaded from the CGGA database (9), which contains glioma data on more than 2000 samples from a Chinese cohort, to supplement or verify the results. Collectively, CGGA samples (n=1018), TCGA tumors (n=689), GTEx normal samples (n=1157), GSE4271 (10–12) and GSE4412 (13) datasets were included in this study. The clinical information of patients included in the TCGA and CGGA data is shown in **Tables 1**, **S1** and **S2**.

**Table 1:** Baseline data of TCGA patients.

Characteristic	Levels	Overall
n		696
WHO grade, n (%)	G2	224 (35.3%)
	G3	243 (38.3%)
	G4	168 (26.5%)
IDH status, n (%)	WT	246 (35.9%)
	Mut	440 (64.1%)
1p/19q codeletion, n (%)	codel	171 (24.8%)
	non-codel	518 (75.2%)
Gender, n (%)	Female	298 (42.8%)
	Male	398 (57.2%)
Race, n (%)	Asian	13 (1.9%)
	Black or African American	33 (4.8%)
	White	637 (93.3%)
Age, n (%)	<=60	553 (79.5%)
	>60	143 (20.5%)
Histological type, n (%)	Astrocytoma	195 (28%)
	Glioblastoma	168 (24.1%)
	Oligoastrocytoma	134 (19.3%)
	Oligodendroglioma	199 (28.6%)
Age, median (IQR)		45 (34, 59)

IDH, Isocitrate dehydrogenase; IQR, interquartile range.

### Expression and genetic alteration analysis of H2B family members

Differential gene expression was analyzed using log2-transformed data from the TCGA dataset and results were visualized using R (version 3.6.3) package ggplot2 (version 3.3.3). All clinical data were enumerated and duplicate samples were removed. H2B protein expression was compared in normal and glioma tissues using the Human Protein Atlas (HPA) database. The Clinical Proteomic Tumor Analysis Consortium (CPTAC) of the ULCAN (14) database was used for analysis of pan-cancer protein expression. H2B alteration types and frequency were evaluated by a quick search of the cBioPortal (15, 16).

### Survival prognosis and relationship with clinical variable analysis

RNA-seq data in level 3 high-throughput sequencing fragments obtained from the TCGA data in the per kilobase per million (HTSeq-FPKM) format were converted to TPM format, and log2 conversion was performed for subsequent analysis. WHO grade, 1p/19q codeletion, IDH status (17), age, gender, and race were included in univariate and multivariate regression models. The mRNA expression data was divided into high- and low-expression groups according to tertiles. Survival data (18) was statistically analyzed using the R survival package (version 3.2-10), including overall survival (OS), disease-free survival (DFS), and progression-free interval (PFI), and results were visualized using the survminer R package (version 0.4.9) (18). CCGA data provided evidence to support a role for H2Bs as prognostic biomarkers of glioma. The timeROC packages (version 0.4) were used to assess the predictive power of H2B molecular expression and time-related outcomes. Decision curve analysis (DCA) was used to evaluate the clinical benefit of risk factors in the multivariate regression analysis.

Logistic analysis was performed using H2BC5, H2BC9, H2BC11, and H2BC21 as binary variables to assess the relationship between these genes and each clinical variable. TCGA and CGGA data were also used to visually analyze the expression of each gene in response to different clinical variables.

### Immune infiltration analysis

The ssGSEA algorithm (19) was used to measure correlations between differentially expressed genes and markers of 24 immune cells (20) using the R GSVA package (version 1.34.0). STROMALScore, ImmuneScore, and ESTIMATEScore algorthims were used to quantify the association between H2BC5, H2BC9, H2BC11, and H2BC21 expression and the abundance of six types of tumor-infiltrating immune cells, CD4+ T cells, CD8+ T cells, B cells, neutrophils, dendritic cells, and macrophages using the estimate (21) packages (version 1.0.13). The TIMER 2.0 database (22–24) was used to further explore the relationship between H2B genes and immune infiltration, tumor purity, and correlation analysis of the corresponding biomarkers. The TISIDB (25) database, which contains multiple cohorts of tumor immune infiltration data, was used to explore the relationship between H2Bs gene and lymphocytes, immune stimulators, immune inhibitors, MHC molecules, chemokines, and chemokine receptors. The association between H2B gene expression and immune subtypes in different human cancers was also investigated. Timer, QUANTISEQ (26), XCELL (27), EPIC (28), and MPCOUNTER (29) algorithms were used to evaluate the relationship between immune infiltration and particular immune cells.

### Functional enrichment analysis

The GENEMANIA (30) database was used to perform a functional analysis of the genes most related to the H2B gene family. Function and pathway enrichment analysis of H2B-encoded proteins was then conducted using the STRING (31) database, which covers more than 24 million proteins from 5,090 organisms, with a minimum required interaction score of ≥0.9 (highest confidence) to construct the PPI network. Using the clusterProfiler (32) (version 3.14.3) package, gene sets divided into H2BC5, H2BC9, H2BC11, and H2BC21 high- and low-expression groups were combined with GO, KEGG, and GSEA (33) logFC values for enrichment pathway analysis. The GOplot (34) (version 1.0.2) package was used to calculate z-score. MSigDB Collections data was used for GSEA analysis with c2.cp.v7.symbols.gmt as the reference, and a nominal false discovery rate (FDR) <0.25 and P-value <0.05 were considered significant enrichment.

### Cell culture and transfection

The human glioblastoma cell lines A172, U-87 MG were purchased from the Cell bank of Chinese Academy of Sciences Shanghai Branch. The cells were cultured in DMEM supplemented with 2 mM L-glutamine and 10% fetal bovine serum (FBS). The cells were incubated in a humidified incubator with 5% CO2 at 37°C. The mRNA sequences and siRNA sequences of H2B in A172 and U87 MG cells are shown in the supplement.

### Cell cycle analysis

A172 and U-87 MG cells were cultured in six-well serum-free culture with 1 × 10^5^ cells and were transfected with H2B siRNA. The cells were collected after 48 h, washed with pre-chilled phosphate-buffered saline (PBS), and then fixed with 70% pre-cooled ethanol at 4°C for more than 2 hours. Samples were stained using Propidium staining. The cell cycle was detected by a Cell Cycle and Apoptosis Analysis Kit (40301ES60; YEASEN, Shanghai, China), and the specific operation method was carried out according to the instructions.

### Methylation analysis of H2B family members

Diseasemeth (35), a database containing aberrant methylomes from human diseases, was used to assess H2B family gene methylation in glioma. The Methsurv (36) database was used to further study the difference in methylation of genes from a different TCGA cohort. Finally, the relationship between the four methyltransferase markers, including DNMT1, DNMT2, DNMT3A, and DNMT3B, and the H2B gene family, was assessed.

### Statistical analysis

H2B gene family expression data was assessed using the Shapiro-Wilk normality test, and the sample did not conform to a normal distribution. The differential gene expression data was accepted by the Wilcoxon rank sum test. We quantitatively analyzed the immunohistochemical images of the HPA database through the “trainable weka segmentation” plugin and “analyze particles” function of iMageJ.

## Results

### Analysis of gene expression and genetic alterations

**Figure 2:**
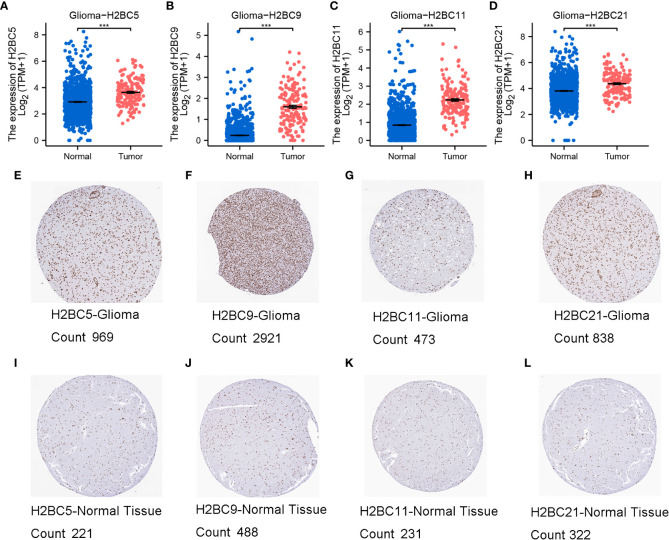
Expression of H2BC5, H2BC9, H2BC11, and H2BC21. **(A–D)** H2BC5, H2BC9, H2BC11, and H2BC21 genes were highly expressed in glioma. H2BC5, H2BC9, H2BC11, and H2BC21 protein expression was higher in **(E–H)** GBM than in **(I–L)** normal tissue using the Human Protein Atlas database (immunohistochemistry) The brown part represents positive cells. “Count” stands for positive cell count. ***P < 0.001.

Using the TCGA database, H2B gene expression was measured in normal and tumor tissues. H2BC1 was not expressed in almost all tumor types (**Figure S1A**), while the other H2B genes were differentially expressed in cholangiocarcinoma, esophageal carcinoma, glioblastoma multiforme (GBM), head and neck squamous cell carcinoma, and cutaneous melanoma (**Figures S1B–Q**). Expression of H2BC5, H2BC9, H2BC11, and H2BC21 was higher in glioma tissues (**Figures 2A–D**). Immunohistochemical data from the HPA database also showed that protein expression of H2B genes was more highly expressed in glioma than normal tissues, and the positive cell count of the pictures by ImageJ made the results more intuitive (**Figures 2E–L**, and **Figures S2A–D**). The ULCAN database was also used to assess H2B protein expression in various types of cancers (**Figures S2E–O**). As shown in **Figure S2I**, H2BC4 was highly expressed in glioblastoma. H2B family genes were relatively conserved, with only H2BC18 and H2BC21 having a slightly higher alteration frequency. Mature B-cell tumors and ovarian epithelial cancers showed the highest frequency of H2B gene alteration, while the alteration frequency in glioma was relatively low (**Figures S3A–Q**). The specific types and sites of H2BC5 genetic alterations are presented in **Figure S3R**. Missense mutation of H2BC5 was the most important type of alteration. **Figure S3S** shows the 3D structure of this gene.

### Prognostic significance of H2B gene expression in glioma

**Figure 3:**
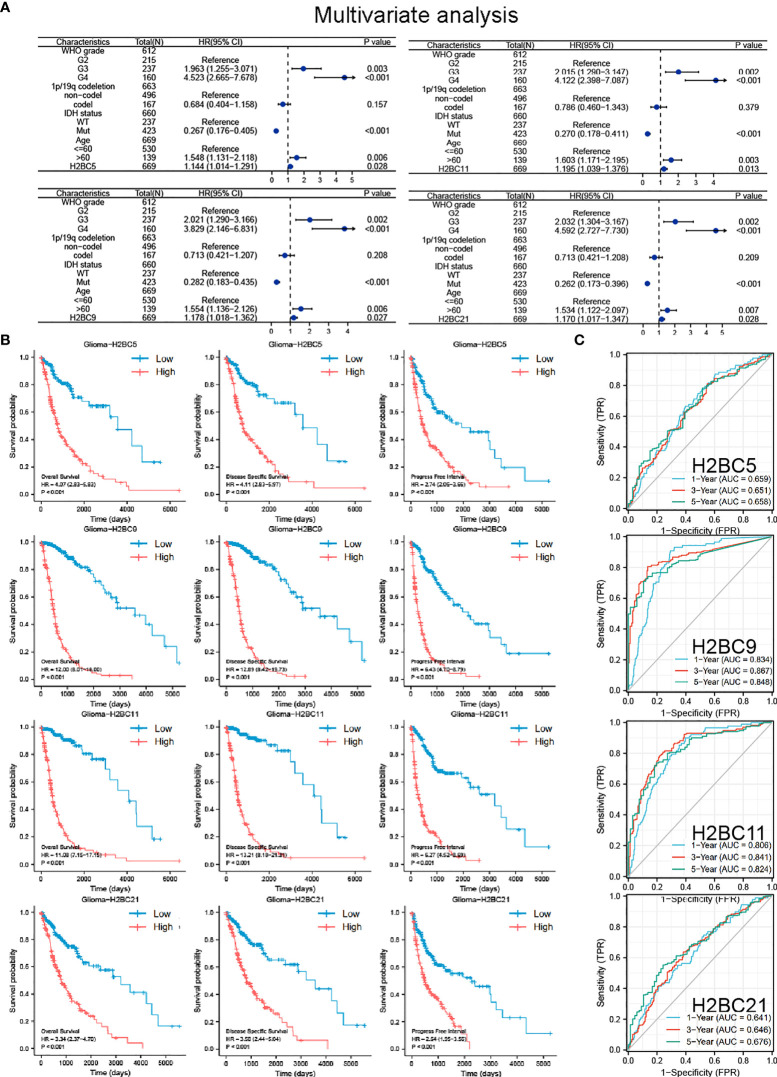
COX regression analysis, Kaplan-Meier survival analysis, and ROC curve of H2BC5, H2BC9, H2BC11 and H2BC21. **(A)** Multivariate Cox regression analyses using TCGA cohorts. Kaplan-Meier survival analysis correlated high expression of H2BC5, H2BC9, H2BC11, H2BC21 with the poor prognosis of OS, DFS, and PFI for glioma patients using the **(B)** TCGA databases. **(C)** ROC curves of glioma overall survival demonstrate the sensitivity and specificity of the predictive power of H2BC5, H2BC9, H2BC11, and H2BC21. P<0.05 is considered statistically significant.

To identify the prognostic value of H2B gene expression in glioma patients, univariate and multivariate Cox regression analyses were performed using the TCGA cohorts (**Figure S4**). Since genes are highly correlated, multivariate regression analysis was conducted separately for each gene along with other patient clinical characteristics to avoid the interference of multi-collinearity (**Figure 3A**). Both univariate and multivariate regression analysis indicated that H2BC5, H2BC9, H2BC11, H2BC21 were risk factors for overall survival of glioma patients, and had a similar impact as WHO grade, IDH status, and age. Other H2B genes were also risk factors in univariate regression analysis, but lost statistical significance in the multivariate regression analysis (**Table S3**).

**Figure 4:**
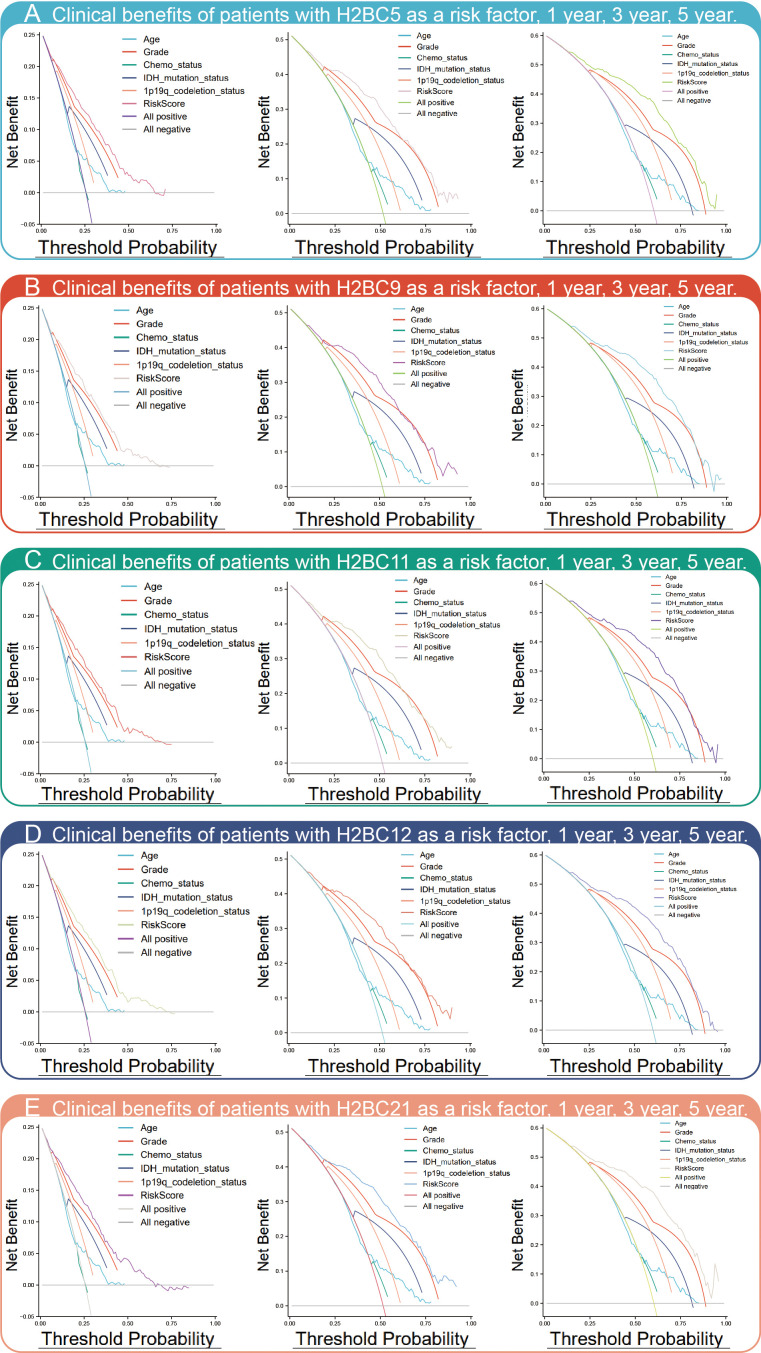
DCA chart of clinical benefits. DCA assesses the clinical benefit (In this study refers to the mortality rate) of each risk factor, including **(A)** H2BC5, **(B)** H2BC9, **(C)** H2BC11, **(D)** H2BC12, and **(E)** H2BC21.

Kaplan-Meier survival analysis showed that high expression of H2BC5, H2BC9, H2BC11, and H2BC21 correlated with a poor OS, DFS, and PFI of glioma patients (**Figure 3B**). These results were verified using the mRNA_693 and mRNA_325 cohort of CGGA (**Figure S4**). The GSE4271 and GSE4412 datasets were used as a supplement to the results and suggested the prognostic value of H2BC5, H2BC9, H2BC21 on astrocytoma (**Figure S4**). Moreover, the ROC curve showed that H2BC5, H2BC9, H2BC11, and H2BC21 all had meaningful predictive power for glioma, especially H2BC9 and H2BC11, which showed excellent sensitivity and specificity in predicting the survival of glioma patients, with an area under the curve (AUC) of >0.8 (**Figure 3C** and **Table 2**). Age is one of the most commonly used clinical prognostic factors, and its sensitivity and specificity for prognostic ability are poorer than those of H2BC5, H2BC9, H2BC11 and H2BC21 (**Figures S5A, B**). Finally, DCA showed that chemotherapy had no obvious benefit on patients in any case, and the expression levels of H2BC5, H2BC9, H2BC11, H2BC12, and H2BC21 correlated with patient outcomes after 1, 3, and 5 years (**Figures 4A–E**). Collectively, these data indicated that H2BC5, H2BC9, H2BC11, and H2BC21 could independently predict glioma prognosis. Abnormal expression of other H2B genes correlated with poor blood cancer, bladder cancer, breast cancer, colorectal cancer, and lung cancer prognoses (**Figures S6A–E**).

**Table 2:** ROC curve of H2B genes.

GENE	TIME	AUC	HR (CI)	SR	BCV	Sensitivity	Specificity	PPV	NPV
H2BC5	1 Year	0.659	0.602-0.716	0.854	3.491716	0.874	0.397	0.199	0.948
	3 Year	0.651	0.593-0.708	0.593	3.604971	0.811	0.451	0.504	0.777
	5 Year	0.658	0.582-0.735	0.454	3.604971	0.783	0.485	0.647	0.65
H2BC9	1 Year	0.834	0.795-0.873	0.854	0.635347	0.908	0.708	0.347	0.978
	3 Year	0.867	0.827-0.908	0.593	0.635347	0.806	0.864	0.803	0.866
	5 Year	0.848	0.799-0.897	0.454	0.521505	0.727	0.879	0.878	0.728
H2BC11	1 Year	0.806	0.762-0.849	0.854	1.466193	0.899	0.603	0.28	0.972
	3 Year	0.841	0.798-0.884	0.593	1.49289	0.808	0.759	0.697	0.852
	5 Year	0.824	0.768-0.881	0.454	1.472237	0.738	0.803	0.818	0.718
H2BC21	1 Year	0.641	0.579-0.702	0.854	4.990272	0.746	0.475	0.196	0.916
	3 Year	0.646	0.588-0.704	0.593	5.158759	0.638	0.617	0.534	0.712
	5 Year	0.676	0.604-0.748	0.454	5.318123	0.553	0.758	0.733	0.584

ROC, Receiver operating characteristic; AUC, Area Under Curve; HR, Hazard Ratio; SR, Survival rate; BCV, The best cut-off value; PPV, Positive predictive value; NPV, Negative predictive value.

### Association between H2B expression and clinical variables

Logistics regression was used to analyze the inference efficacy of a single gene on clinical variables and evaluate the relationship between H2BC5, H2BC9, H2BC11, and H2BC21 and clinical variables. Increased expression of H2B genes in glioma tissue was significantly correlated with WHO grade, 1p/19q codeletion, IDH status, age, and outcome of primary therapy (**Table 3**).

**Table 3:** Logistic regression of H2B genes.

Gene	Characteristics	Total(N)	Odds Ratio(OR)	P value
H2BC5	WHO grade (G3&G4 vs. G2)	635	3.245 (2.311-4.588)	< 0.001
	1p/19q codeletion (non-codel vs. codel)	689	2.001 (1.407-2.866)	< 0.001
	IDH status (Mut vs. WT)	686	0.327 (0.235-0.453)	< 0.001
	Age (>60 vs. < =60)	696	2.174 (1.489-3.203)	< 0.001
	Primary therapy outcome (PR&CR vs. PD&SD)	462	0.569 (0.390-0.827)	0.003
H2BC9	WHO grade (G3&G4 vs. G2)	635	7.496 (5.164-11.049)	< 0.001
	1p/19q codeletion (non-codel vs. codel)	689	8.654 (5.588-13.876)	< 0.001
	IDH status (Mut vs. WT)	686	0.047 (0.030-0.073)	< 0.001
	Age (>60 vs. < =60)	696	4.212 (2.795-6.490)	< 0.001
	Primary therapy outcome (PR&CR vs. PD&SD)	462	0.532 (0.355-0.789)	0.002
H2BC11	WHO grade (G3&G4 vs. G2)	613	7.895 (5.387-11.761)	< 0.001
	1p/19q codeletion (non-codel vs. codel)	664	15.061 (9.075-26.539)	< 0.001
	IDH status (Mut vs. WT)	661	0.070 (0.045-0.105)	< 0.001
	Age (>60 vs. < =60)	670	3.094 (2.078-4.680)	< 0.001
	Primary therapy outcome (PR&CR vs. PD&SD)	444	0.598 (0.401-0.886)	0.011
H2BC21	WHO grade (G3&G4 vs. G2)	613	2.619 (1.863-3.701)	< 0.001
	1p/19q codeletion (non-codel vs. codel)	664	2.207 (1.541-3.186)	< 0.001
	IDH status (Mut vs. WT)	661	0.354 (0.254-0.492)	< 0.001
	Age (>60 vs. < =60)	670	2.230 (1.518-3.310)	< 0.001

HR, Hazard Ratio; IDH, Isocitrate dehydrogenase; PR, Partial response; CR, complete response; PD, progression disease; SD, stable disease.

**Figure 5:**
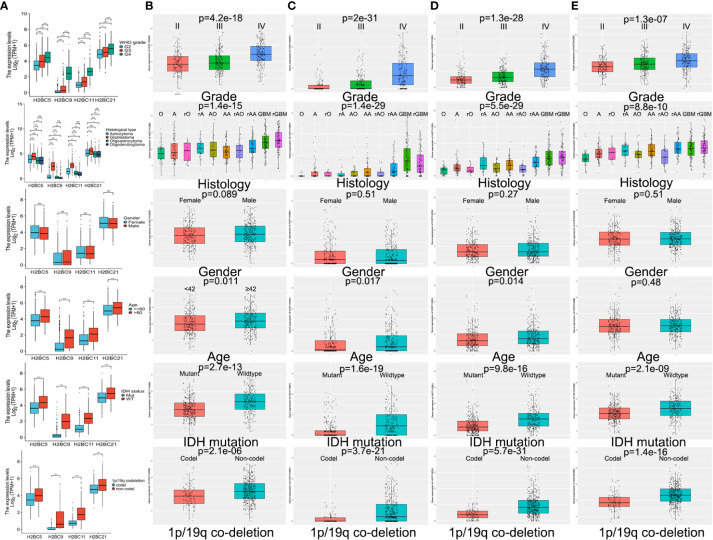
Influence of various factors on gene expression using TCGA and CGGA data. Influence of WHO grade, histology, IDH status, 1p/19q codeletion, gender, and age on H2B gene expression in **(A)** TCGA and **(B–E)** CGGA. P<0.05 is considered statistically significant. *P < 0.05, **P < 0.01, ***P < 0.001, ns, no significance.

TCGA and CGGA data were used to explore the relationship between H2B gene expression and various clinical factors. Findings showed that a rise in the degree of glioma malignancy correlated with increased expression of H2B genes. No difference in H2B expression was shown by gender. The relationship between H2BC21 expression and age differed slightly between the CGGA and TCGA databases, which may be caused by discrepancies in age stratification. Glioma with wild-type IDH had higher H2B gene expression, while those with 1p/19q codeletion had lower H2B gene expression (**Figures 5A–E**). Some results indicate that H2Bs gene expression was higher in recurrent glioma (**Figure S7**), however, more data is needed to confirm these findings.

### Immune infiltration analysis of H2B genes

**Figure 6:**
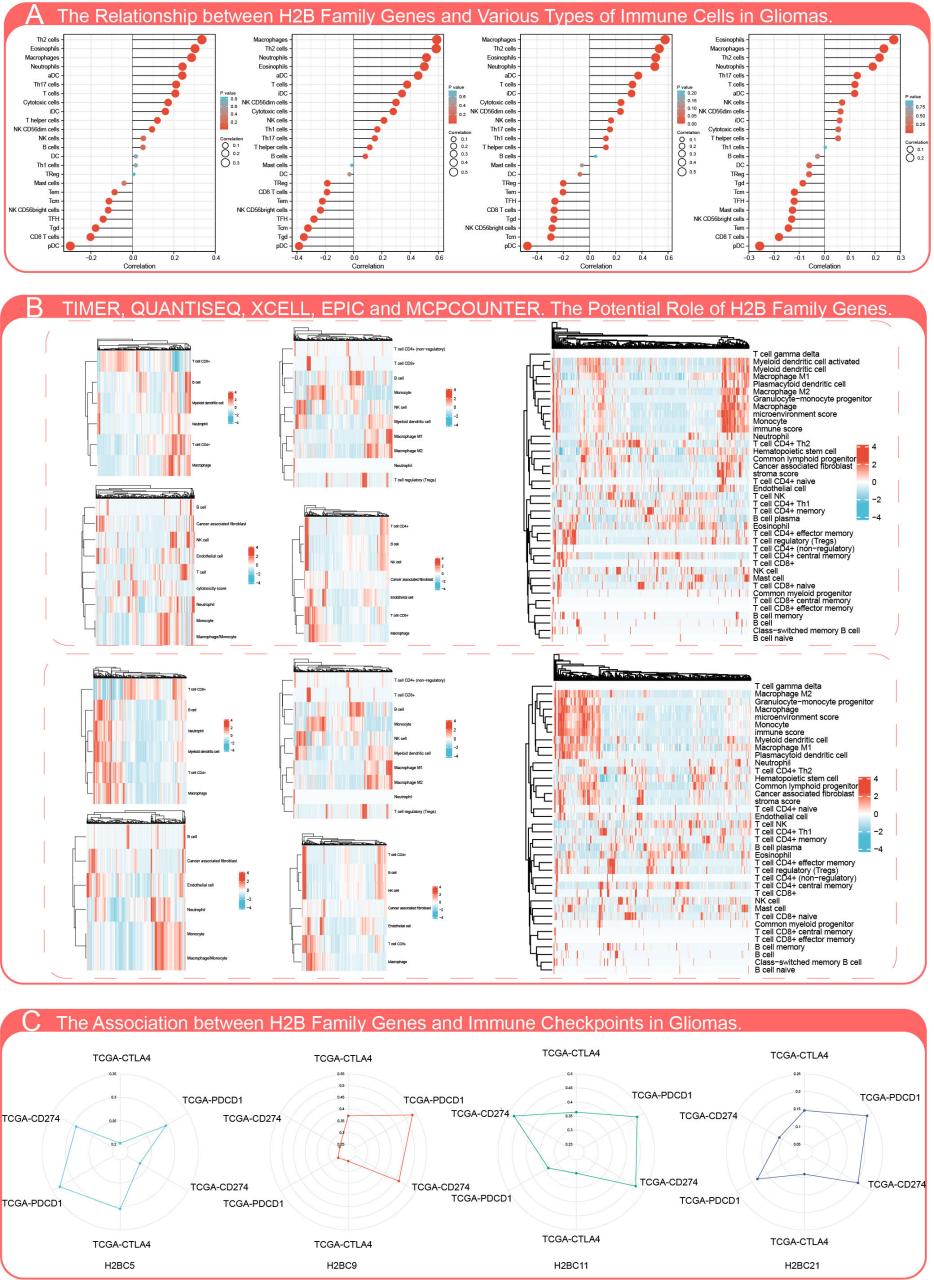
Influence of H2B gene expression on immune cell infiltration. **(A)** Correlation analysis of H2BC5, H2BC9, H2BC11, and H2BC21 (from left to right) gene expression and infiltration of various types of immune cells. **(B)** Timer, QUANTISEQ, XCELL, EPIC, MPCOUNTER algorithms were used to analyze the correlation between H2B gene expression and immune cell infiltration in low-grade (top) and malignant glioma (bottom). **(C)** Correlation analysis of H2BC5, H2BC9, H2BC11, and H2BC21 gene expression and the immune checkpoint biomarkers, CTLA4, PDCD1, and CD274.

The influence of H2BC5, H2BC9, H2BC11 and H2BC21, which can be regarded as independent prognostic factors, on immune infiltration was evaluated in 24 kinds of immune cells. H2BC9 and H2BC11 were positively correlated with macrophages, Th2 cells, neutrophils, and eosinophils, and negatively correlated with pDC cells (**Figure 6A**). Timer, QUANTISEQ, XCELL, EPIC, and MPCOUNTER algorithms were used to evaluate the relationship between H2B family genes and infiltration of different immune cells, and the results showed similar trends (**Figure 6B**). Correlation between H2B family genes and lymphocytes, immune stimulators, immune inhibitors, MHC molecules, chemokines, and chemokine receptors biomarkers in various tumor types (**Figures S8A–L**), was partially verified using GBM and LGG cohorts from TCGA (**Table S4–7**). H2BC5, H2BC9, H2BC11, and H2BC21 were closely related to the immune subtype depleted lymphocytes. The correlation between immune checkpoints, which compared data from TCGA and CGGA, was also visualized (**Figure 6C**).

**Figure 7:**
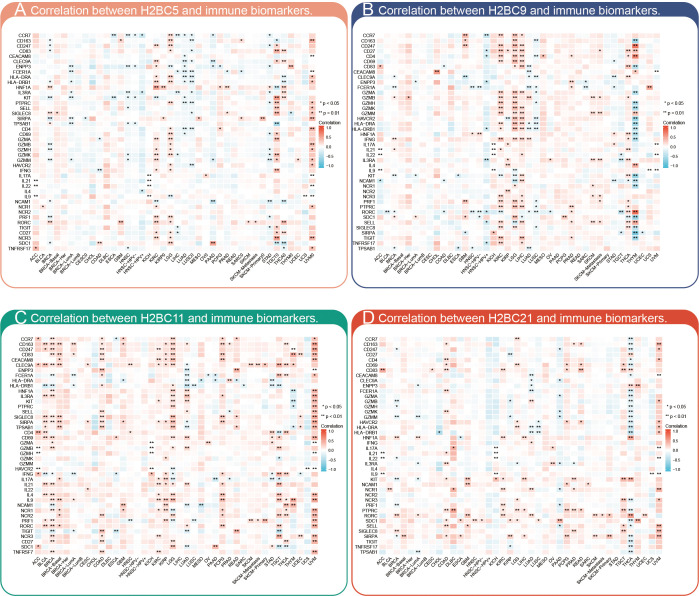
Pan-cancer analysis of immune infiltration. Pan-cancer analysis of the correlation between **(A)** H2BC5, **(B)** H2BC9, **(C)** H2BC11, and **(D)** H2BC21 expression and multiple immune cell biomarkers. *P < 0.05, **P < 0.01.

**Figure 8:**
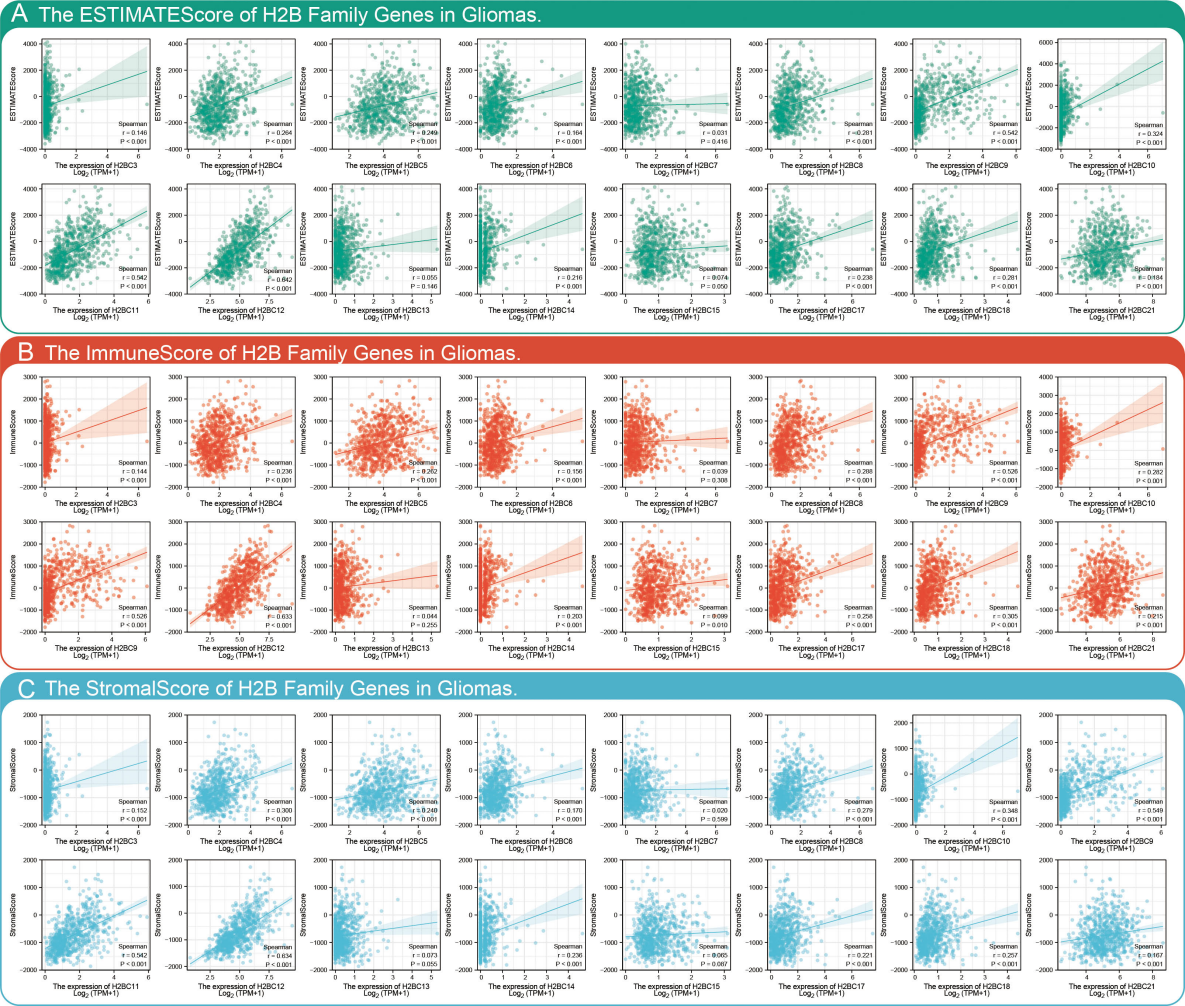
Combining STROMALScore, ImmuneScore, and ESTIMATEScore to evaluate the relationship between H2B gene expression and tumor immune cell infiltration. Immune score of H2B genes in glioma based on the **(A)** STROMALScore, **(B)** ImmuneScore, and **(C)** ESTIMATEScore algorithm. r>0.3 means low correlation, r>0.5 means moderate correlation. P<0.05 is considered statistically significant.

The relationship between H2BC5, H2BC9, H2BC11, and H2BC21 and biomarkers of different immune cells in various cancers is shown in **Figures 7A–D**. The STROMALScore, ImmuneScore, and ESTIMATEScore algorithms also suggested that H2BC9, H2BC11 and H2BC12 were positively correlated with immune infiltration (**Figures 8A–C**), helping to fill the research gap (7). Notably, H2BC5, H2BC9, H2BC11, and H2BC21 were highly expressed in patients who did not respond to atezolizumab (anti-PDL1) immunotherapy for urothelial cancer (**Figure S9**).

### Functions and pathways of H2B genes in glioma

**Figure 9:**
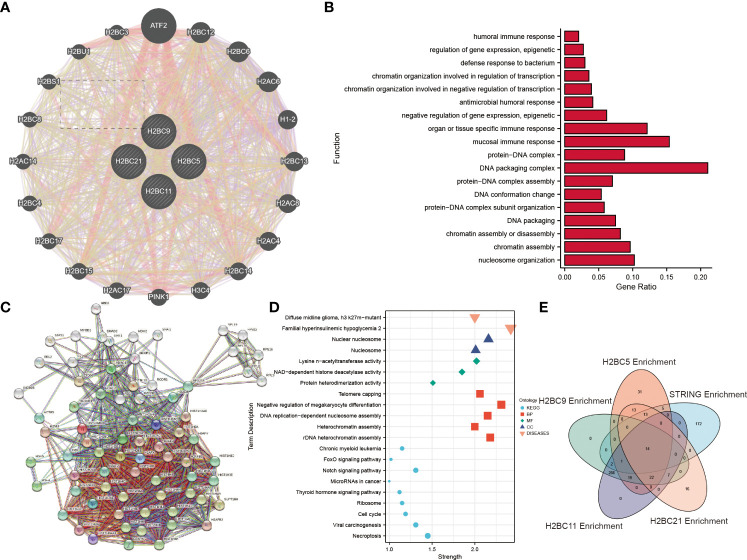
Enrichment analysis and protein-protein interaction (PPI) network of H2B genes. **(A)**. The 20 genes from the GENEMANIA database that were most related to H2BC5, H2BC9, H2BC11 and H2BC21 were enriched and analyzed. **(B)** Enrichment analysis results are displayed in a histogram. **(C)** A protein-protein interaction network for the 50 proteins most directly related to H2BC5, H2BC9, H2BC11 and H2BC21, and 20 indirectly related proteins, was constructed using the STRING database. **(D)** Some of the enrichment analysis results were displayed in dot plots. **(E)** Overlapping pathways and/or functions is shown using a Venn diagram.

GENEMANIA data was used to explore the functions of 20 genes that correlated with H2BC5, H2BC9, H2BC11, and H2BC21 expression. H2BC11, H2BC21, H2BC12, and H2BS1 were all involved in humoral, mucosal, and organ- or tissue-specific immune responses (**Figures 9A, B**). To further explore the functions and pathways of proteins encoded by H2BC5, H2BC9, H2BC11, and H2BC21 in glioma, 74 proteins that were strongly related to H2B, including 50 directly related and 20 indirectly related proteins obtained from the STRING database, were used to construct a PPI network. Some of these results are visualized in **Figures 9C, D** and the complete results are shown in **Table S8**. The enrichment analysis showed that H2B genes participate in many pathological processes of glioma, including necroptosis, FOXO and Notch signaling, and the cell cycle.

**Figure 10:**
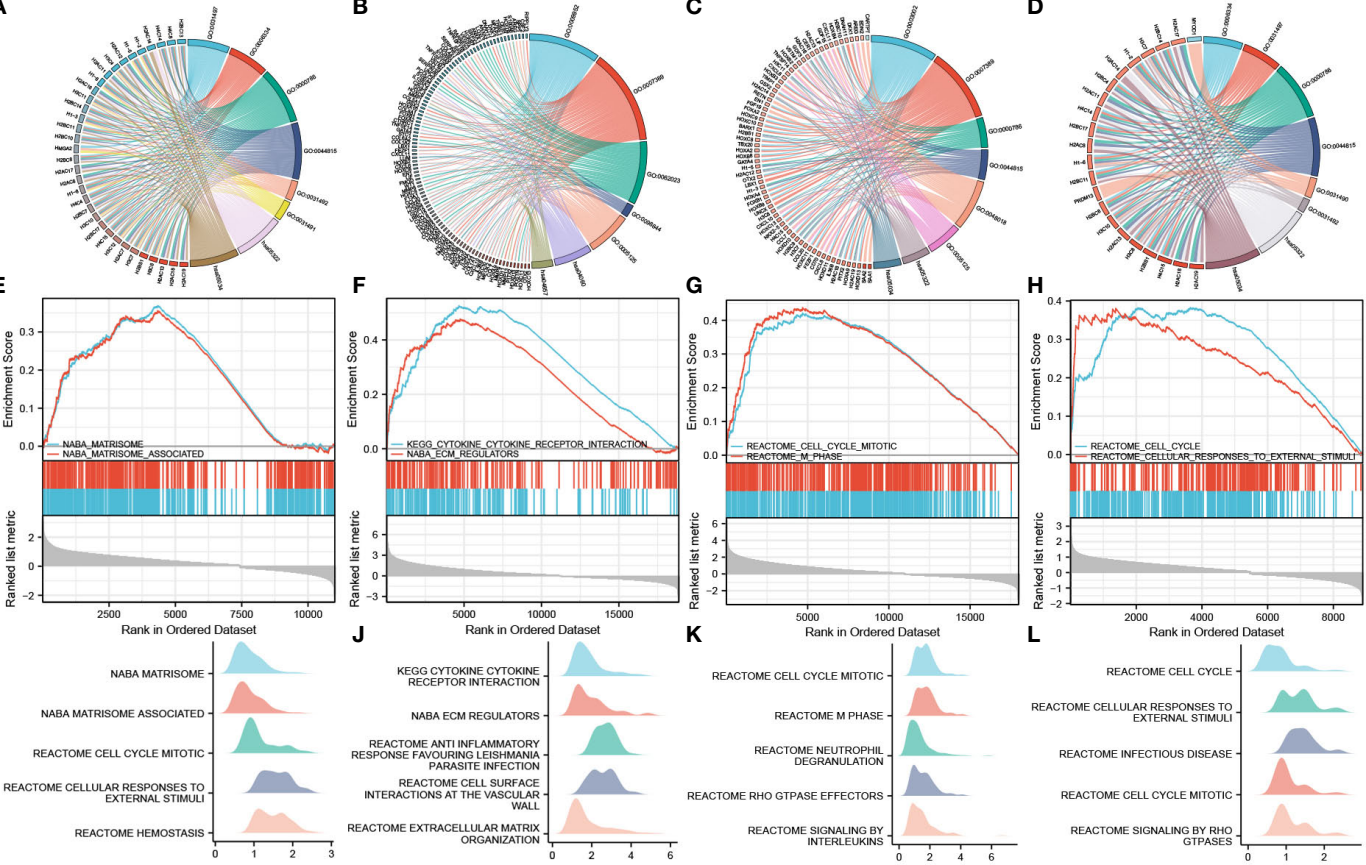
Combined GO and KEGG analysis of logFC value and GSEA analysis. The left side of the chord diagram is the genes used for the enrichment analysis, and the right side is the enrichment analysis result. Different result items are linked to corresponding genes and are distinguished by different colors. **(A–D)** H2BC5, H2BC9, H2BC11, and H2BC21 were divided into high- and low-expression groups, and the logFC value was combined for GO and KEGG analysis. **(E–H)** H2BC5, H2BC9, H2BC11, and H2BC21 were divided into high- and low-expression groups, and GSEA analysis was performed. **(I–L)** Other GSEA analysis results are shown in mountain maps.

LogFC values were combined to perform enrichment analysis on H2BC5, H2BC9, H2BC11, and H2BC21, and similar results were seen in Venn diagrams (**Figure 9E**). A total of 14 functions or pathways were observed, including transcriptional misregulation of cancer (hsa05202), chromatin silencing at rDNA (GO:0000183), regulation of megakaryocyte differentiation (GO:0045652), and regulation of myeloid cell differentiation (GO:0045637) (**Figures 10A–D**), which likely contribute to the pathological process of cancer. H2BC5, H2BC9, H2BC11, and H2BC21 were divided into high- and low- expression groups and analyzed by GSEA (**Figures 10E–H**), and the results were visualized using mountain maps (**Figures 10I–L**). In addition to the histone-specific functions, results from Reactome indicated that these genes were involved in cell cycle pathways, neutrophil degranulation, and interleukin signaling, which may play a key role in cancer progression.

### Inhibition of the H2B family genes affects glioma by regulating the cell cycle

**Figure 11:**
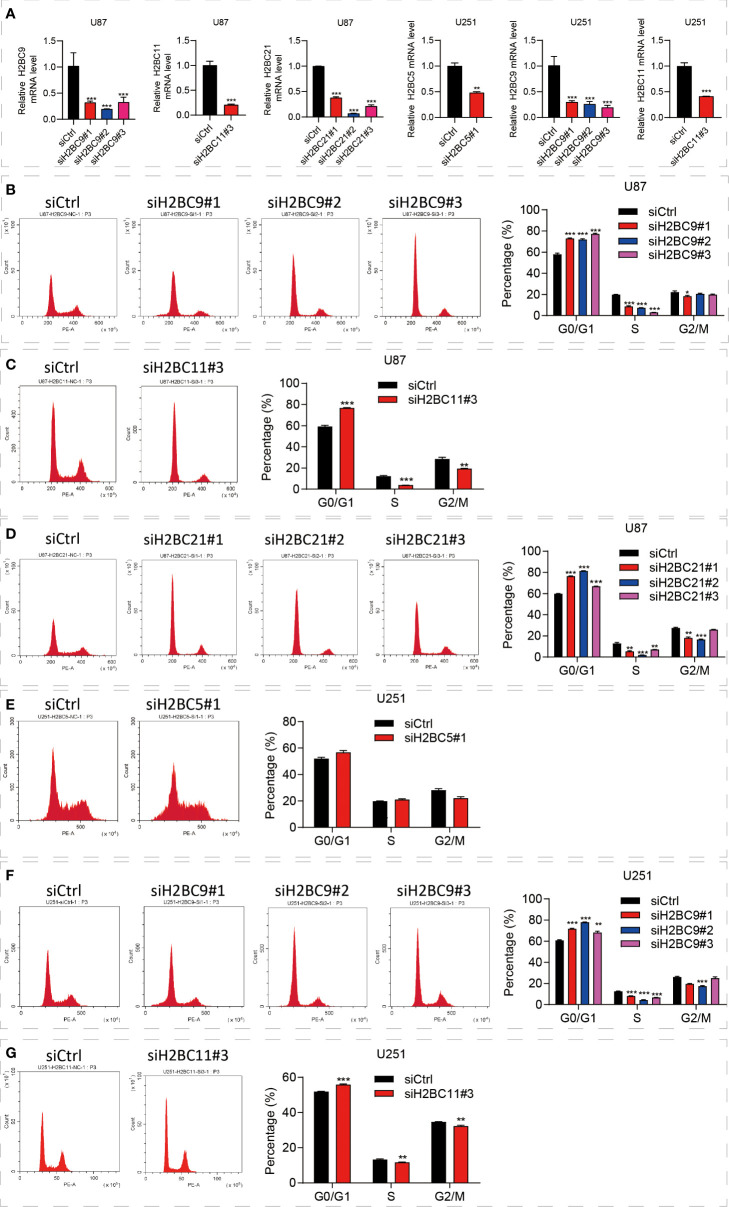
Knockdown of the H2B gene significantly affected the cell cycle. **(A)** Downregulated H2BC5, H2BC9, H2BC11 and H2BC21 expression levels upon transfection each siH2Bs in U87 MG and U153 cell lines separately. **(B–G)** Flow cytometry was used to detect the cell cycle distribution of U87 and U251 cell lines. **P < 0.01, ***P < 0.001.

The above bioinformatics analysis indicated that the H2B family genes were significantly enriched in the cell cycle pathway. Since the cell cycle of glioma is closely related to prognosis, we proposed that the poor prognosis of glioma is related to the influence of H2B on the cell cycle (37, 38), and carried out further experimental verification. To identify the functional role of H2BC5, H2BC9, H2BC11 and H2BC21 in gliomas, the A172 and U-87 MG cell lines were treated with siRNA against the H2B genes (**Figure 11A**). The results suggested that U87 cells revealed G0/G1 cell cycle arrest after H2BC9, H2BC11, H2BC21 knockdown (**Figures 11B–D**) and U251 cells revealed G0/G1 cell cycle arrest after H2BC5, H2BC9 and H2BC11 knockdown (**Figures 11E–G**). These data imply that high expression of H2B family genes in glioma patients accelerates the cell cycle, thereby contributing to glioma progression

### The methylation of H2B family genes in glioma

**Figure 12:**
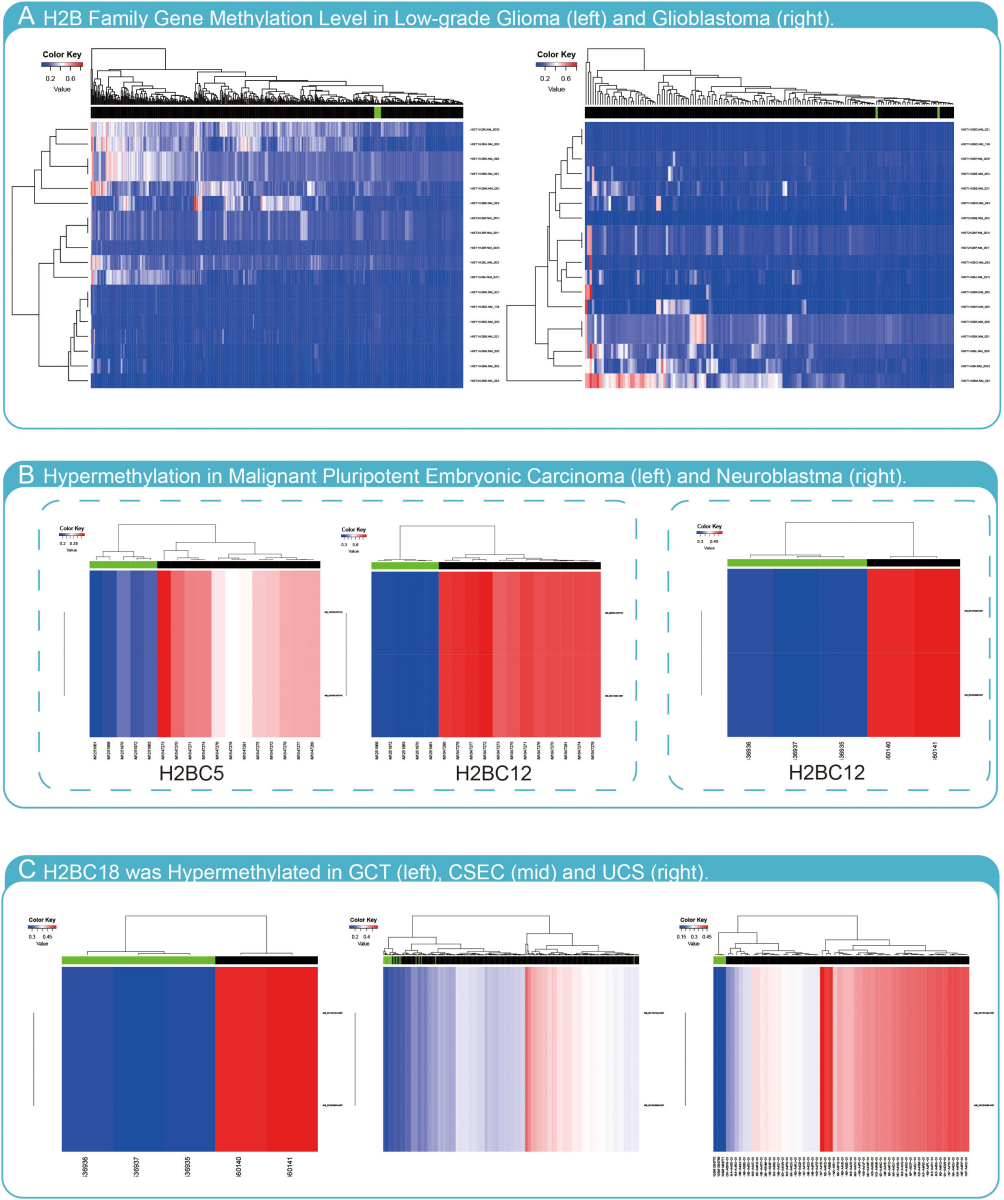
Methylation analysis of H2B genes. **(A)** H2B genes have low methylation levels in LGG and GBM. **(B)** H2BC5 was hypermethylated in malignant pluripotent embryonic carcinoma, and H2BC12 was hypermethylated in malignant pluripotent embryonic carcinoma and neuroblastoma. **(C)** H2BC18 was hypermethylated in germ cell tumors (GCT), cervical squamous cell carcinoma and endometrial adenocarcinoma (CSEC), and uterine carcinosarcoma (UCS).

To determine whether methylation of H2B genes impacts glioma progression, H2B gene expression was evaluated using the Meth database. The results indicated that most H2B genes had no hypermethylation status in glioma (**Figure 12A**). However, H2BC5 and H2BC12 were found to be hypermethylated in malignant pluripotent embryonic carcinoma (**Figure 12B**), and H2BC12 was also shown to be hypermethylated in neuroblastomas. In addition, H2BC18 had a hypermethylated status in germ cell tumors (GCT), cervical squamous cell carcinoma and endometrial adenocarcinoma (CSEC), and uterine carcinosarcoma (UCS) (**Figure 12C**). The methylation status of H2BC5, H2BC9, H2BC11, and H2BC21 in different glioma cohorts was evaluated in the Methsurv database (**Figures S10A–H**). The correlation between these genes and methyltransferase markers was weak (**Figure S10I**). Thus, findings suggest that the methylation level of H2B genes was likely not enough to affect glioma development.

## Discussion

H2B genes, usually located in the nucleus, are some of the core members of the 22-member histone nucleosome, with the remaining members being H2A, H3, and H4. As a result, the H2B family is often called H2B Histone family with H2BC5 defined as Histone Cluster 1 H2B Family Member D (HIST1H2BD).

Oncohistones have become a common area of study. H3K27M (histone H3Lys27-to-Met missense mutation) and H3G34V/R (H3 Glycine 34 to Valine/Arginine) were initially found in diffuse intrinsic pontine glioma (39–41), and H3K36M (H3 Glycine 34 to Valine/Arginine) was associated with chondroblastoma (42) and head and neck squamous cell carcinoma (43, 44). H2B has three reported mutations, H2BG53D (45, 46), H2BE76K (45) and H2BE113K (45). Indeed, the mutation frequency of individual H2B genes is 0.2 - 0.3%. Prior studies reported (45) that the five most common cancers with H2B mutations are endometrial cancer (13.8%), bladder urothelial cancer (13.4%), cervical squamous cell carcinoma (10.8%), head and neck squamous cell carcinoma (10.3%) and esophageal squamous cell carcinoma (9.5%). These results differ slightly from the results of this study potentially because they each rely on different datasets. Pan-cancer prognosis analysis of H2B gene expression in this study showed that high expression of multiple H2B genes correlated with poor bladder cancer prognosis potentially as a result of an H2B gene mutation.

From a macroscopic perspective, common clinical features such as age, radiotherapy status, chemotherapy status, and tumor recurrence are common clinical prognostic factors (47), and their greatest advantage is that this information is readily available. From a microscopic perspective, molecular biomarkers play an important role in providing auxiliary and defining diagnostic information (6). H2B family genes, especially H2BC5 and H2BC9, as molecular biomarkers, have high specificity and sensitivity for the prognosis of glioma patients, and are significantly correlated with glioma grade, age and other factors, showing a high research potential.

Monoubiquitination of H2B may cause the chromosome instability (CIN) phenotype associated with mitotic chromatin compaction defects (48), a significant feature of oncogenesis associated with poor outcomes. Thus, H2B is involved in many pathological processes of cancer and is linked to a poor prognosis. As a result, it is critical to study the ability of H2B family genes to predict cancer prognosis using bioinformatics.

Along with TCGA, CGGA, and multiple GEO data cohorts, univariate and multivariate regression, KM survival, ROC and DCA, and logistics analyses were conducted to determine the relationship between H2B genes and multiple cancers, especially glioma. Several algorithms were used to make a correlation between H2B family genes and immune cell biomarkers and investigate immune infiltration in glioma and other cancers. Enrichment analysis of H2BC5, H2BC9, H2BC11, and H2BC21 was also conducted to understand how the H2B gene is involved in the pathological progression of glioma.

Univariate and multivariate Cox analysis showed that H2BC5, H2BC9, H2BC11, and H2BC21 are high-risk factors for glioma. K-M analysis also showed that high H2B expression correlates with poor prognosis in patients with this disease. Several studies have shown that H2BC5, H2BC9, and H2BC11 are independent prognostic factors of cervical cancer (49), and H2BC12 is an independent prognostic factor for low-grade glioma (7). ROC analysis showed that H2BC9 and H2BC11 AUC values at 1, 3, and 5 years were both >0.8, suggesting that these genes are indicators of patient survival. Results of the DCA curve showed that H2BC5, H2BC9, H2BC11, H2BC12, and H2BC21 expression correlated with positive clinical outcomes at one, three, and five years. As a result, further research on H2B genes may have great value for the clinical prognosis of glioma patients.

The immune-related interaction mechanism plays an important role in the occurrence and development of glioma, and immunotherapy is considered to have important research value in glioma. This study used multiple algorithms to assess the correlation between H2B gene expression and immune cell infiltration and found that H2B family genes, especially H2BC9 and H2BC11 are closely related to the immune system of glioma patients. First, we used the ssGSEA algorithm to assess immune cell infiltration in gliomas and found that H2BC9 and H2BC11 were positively correlated with influx of macrophages, Th2 cells, neutrophils and eosinophils. Tumor-associated macrophages can promote the proliferation of gliomas through multiple pathways, including promoting an immunosuppressive environment (50, 51) or increasing calcium levels (52). In the current study, neutrophils were also considered to be a risk factor for the development of gliomas (53, 54). On the other hand, H2BC9 and H2BC11 were inversely associated with pDC cells, which promoted the recruitment of regulatory T cells into the tumor microenvironment and resulted in immunosuppression and tumor growth (55). Therefore, these findings powerfully indicate that H2BC9 and H2BC11 play a special role in the development of glioma and the formation of an immunosuppressive environment. In order to further analyze the correlation between H2B family genes and various types of tumor-infiltrating immune cells, we focused on the correlation between their mRNA expression levels and immune marker sets of corresponding immunocytes. The analysis data showed that H2BC9 mRNA expression level was positively correlated with the mRNA expression levels of marker genes in neutrophils, eosinophils, M1 macrophages T cells, myeloid-derived suppressor cell (MDSC) in glioma, and negatively correlated with the mRNA expression levels of marker genes in NKT cells and cDC2s cells. MDSC cells play a key role in mediating tumor immune escape, including decreased phagocytic ability, and increased ability to induce apoptosis in activated lymphocytes (56). NKT cells are one of the keys to killing gliomas and are the focus of research in immunotherapy (57). There is no doubt that the H2B family of genes is involved in tumor immune escape and in some way reduces the ability of, for example, NKT cells to kill glioma cells. Next, by analyzing the correlation of H2B family genes with immune checkpoint biomarkers and immune regulators, we found that H2BC5 and H2BC11 are closely related to immunoinhibitor. H2BC9 and H2BC11 positively correlate with the immune checkpoint PDCD1, which mediates inhibitory pathways that are exploited by tumors to mitigate antitumor immunity and escape destruction by the immune system, thereby promoting tumor survival (58). H2BC5, H2BC9, H2BC11 and H2BC21 showed strong positive correlation with various immune markers, such as IL10 and CD70. The expression level of IL-10 in glioma patients was higher than that in the normal population, and the expression level increased significantly with the increase of the malignant degree of glioma (59). Acuner-Ozbabacan et al. (60) found that IL-10 deficiency leads to the secretion of pro-inflammatory cytokines, which induces and inhibits the body’s anti-tumor immunity, thereby promoting tumor growth. High expression of CD70 is an independent prognostic risk factor for glioma (61). Wischhusen et al. (62) found that CD70 selectively induces CD8+ T cell death to participate in immunosuppression and promote tumor progression. They are one of the reasons for the poor prognosis of glioma caused by high expression of H2BC9 and H2BC11. Besides, The algorithm of the estimate package showed that the expression of H2B family genes, especially H2BC9, H2BC11 and H2BC12 was positively correlated with the presence of stromal cells in glioma, the level of immune cells and the purity of tumor cells. Taken together, these results demonstrate that the H2B family genes are involved in the formation of the glioma immunosuppressive environment and promote the generation and development of gliomas through immune pathways, resulting in poor prognosis in glioma patients. At the same time, it also has the effect of regulating the tumor microenvironment, and has the potential to become a new target for the treatment of glioma. Considering the interaction between H2B family genes and various immune regulators, we believe that H2B family genes may have a broader role in immunity, which will be the direction of future research.

Enrichment analysis results also indicated that enriched pathways are associated with the type of immune response. Thus, H2B genes may suppress the immune response through lymphocyte depletion. In addition, increasing evidence (63–65) suggests that tumor-associated macrophages play a critical role in tumor progression and metastasis. Enrichment analysis of GO, KEGG, and GSEA revealed possible pathways by which H2B genes participate in tumor progression. Functions and pathways of note include but are not limited to transcriptional misregulation in cancer, chromatin silencing at rDNA, regulation of megakaryocyte differentiation, cell cycle, neutrophil degranulation, and interleukin signaling. The cell cycle is closely related to the prognosis of glioma, so we put forward the hypothesis that H2B affects the cell cycle and leads to the poor prognosis of H2B. Next, we used siH2B to further verify that knocking down the H2B gene can cause the cell cycle to stay in the G0/G1 phase through cell experiments. This experimental result confirms our hypothesis.

Compared with previous studies on glioma biomarkers, this study has the advantage of using a richer bioinformatic analysis method including pan-cancer analysis. First, this study adds DCA curves to assess the benefit of each biomarker in glioma prognosis, a method not used by Liu et al (7). Then, compared to Liu et al.’s study (66), more practical immunological analysis methods such as STROMALScore, ImmuneScore and ESTIMATEScore algorithms were also used in this study. Pan-cancer analysis is also an important part of this article. In previous studies such as hu et al. (67) and qu et al. (68), they discussed the relationship between biomarkers and glioma from the perspective of immunity and methylation, respectively. But the fly in the ointment is the lack of a pan-cancer analysis, and the lack of experimental data to draw conclusions. The study of Piezo1 gene upregulation as a prognostic biomarker in glioma by Qu et al (69). has important clinical implications. Through comparison, it is found that the H2B family gene and Piezo1 gene have many similar characteristics, such as high expression of the gene, which predicts the poor prognosis of glioma, but the current research on the H2B family gene is still insufficient. The advantage of this study is that it used multiple data sets, and the results can be mutually confirmed, which provides a reference for the future research direction of H2B family genes.

The current study has several limitations. Expression of H2B gene mRNA was studied and while protein expression from the HPA database was used as a reference, the correlation between mRNA and protein levels requires further research and verification. Moreover, the role of H2B gene expression in tumors and their value as potential targets for anti-tumor therapy need additional study.

In summary, an initial pan-cancer analysis of H2B gene expression showed a significant clinical correlation between H2B gene expression and cancer prognosis, immune cell infiltration, and immune markers. These data aid the current understanding of H2B gene function during tumorigenesis. The analysis of H2B family gene expression in glioma showed that H2BC5, H2BC9, H2BC11, and H2BC21 can be used as independent predictive factors for glioma prognosis, and overexpression of H2BC9 and H2BC11 may lead to tumor deterioration through the immune system.

## Data availability statement

The datasets presented in this study can be found in online repositories. The names of the repository/repositories and accession number(s) can be found in the article/**Supplementary Material**.

## Ethics statement

Ethical review and approval was not required for the study on human participants because all the clinical data of patients in this study were obtained from public databases. Written informed consent for participation was not required for this study in accordance with the national legislation and the institutional requirements.

## Author contributions

JNJ conceived and designed the work. JNJ, ZCH and XKW acquired, analyzed, and interpreted the data. JNJ and ZCH drafted and revised the article. XCZ and SRW researched the literature, and contributed to figures and tables. YLC supervised the study and sought for foundation support. All authors contributed to the article and approved the submitted version.

## Funding

This work was supported by the National Science and Technology Research Project of Traditional Chinese Medicine [CZY-KJS-2021-017], Henan Province Epidemic Prevention and Control Scientific Research Project [No. 211100310600],2022 Henan Province Key R&D Project [221111310500].

## Acknowledgments

We thank “Charlesworth Group’s author services” for its language editing of the manuscript. We truly appreciate all the medical workers fighting against the COVID-19 pandemic.

## Conflict of interest

The authors declare that the research was conducted in the absence of any commercial or financial relationships that could be construed as a potential conflict of interest.

## Publisher’s note

All claims expressed in this article are solely those of the authors and do not necessarily represent those of their affiliated organizations, or those of the publisher, the editors and the reviewers. Any product that may be evaluated in this article, or claim that may be made by its manufacturer, is not guaranteed or endorsed by the publisher.
