# Supplementary material for: H2B gene family: A prognostic biomarker and correlates with immune infiltration in glioma
Source: Front Oncol. 2022 Oct 25;12:966817. doi: 10.3389/fonc.2022.966817 (PMC9641242; doi:10.3389/fonc.2022.966817)
Supplement: Supplementary file 1 [file DataSheet_1.docx]

Supplement Table 1. Baseline data for patients in the CGGA mRNA_325 cohort.

| Characteristic | levels | Overall |
| --- | --- | --- |
| n |  | 325 |
| PRS_type, n (%) | Primary | 229 (71.3%) |
|  | Recurrent | 62 (19.3%) |
|  | Secondary | 30 (9.3%) |
| Histology, n (%) | A | 46 (14.3%) |
|  | AA | 38 (11.8%) |
|  | AO | 12 (3.7%) |
|  | GBM | 85 (26.5%) |
|  | O | 48 (15%) |
|  | rA | 10 (3.1%) |
|  | rAA | 24 (7.5%) |
|  | rGBM | 24 (7.5%) |
|  | rO | 4 (1.2%) |
|  | sGBM | 30 (9.3%) |
| Grade, n (%) | WHO II | 103 (32.1%) |
|  | WHO III | 79 (24.6%) |
|  | WHO IV | 139 (43.3%) |
| Gender, n (%) | Female | 122 (37.5%) |
|  | Male | 203 (62.5%) |
| Age, n (%) | ＜43 | 167 (51.4%) |
|  | ≥43 | 158 (48.6%) |
| IDH_mutation_status, n (%) | Mutant | 175 (54%) |
|  | Wildtype | 149 (46%) |
| 1p19q_codeletion_status, n (%) | Codel | 67 (21.1%) |
|  | Non-codel | 250 (78.9%) |

PRS: Primary, Recurrent, Secondary; A: Astrocytoma; AA: Anaplastic astrocytoma; AO: Anaplastic oligodendroglioma; GBM: Glioblastoma multiforme; rA: Recurrent astrocytoma; rAA: Recurrent anaplastic astrocytoma; rGBM: Recurrent glioblastoma multiforme; rO: Recurrent oligodendroglioma; sGBM: Secondary glioblastoma multiforme; IDH: Isocitrate dehydrogenase

Supplement Table 2. Baseline data for patients in the CGGA mRNA_693 cohort.

| Characteristic | levels | Overall |
| --- | --- | --- |
| n |  | 693 |
| PRS_type, n (%) | Primary | 422 (60.9%) |
|  | Recurrent | 271 (39.1%) |
| Histology | A | 85(12.27%) |
|  | AA | 82(11.83%) |
|  | AO | 46(6.64%) |
|  | AOA | 16(2.31%) |
|  | GBM | 140(20.20%) |
|  | O | 45(6.49%) |
|  | OA | 8(1.15%) |
|  | rA | 34(4.91%) |
|  | rAA | 70(10.10%) |
|  | rAO | 36(5.19%) |
|  | rAOA | 5(0.72%) |
|  | rGBM | 109(15.73%) |
|  | rO | 15(2.16%) |
|  | rOA | 1(0.14%) |
| Grade, n (%) | WHO II | 188 (27.2%) |
|  | WHO III | 255 (36.8%) |
|  | WHO IV | 249 (36%) |
| Gender, n (%) | Female | 295 (42.6%) |
|  | Male | 398 (57.4%) |
| Age, n (%) | ＜43 | 343 (49.6%) |
|  | ≥43 | 349 (50.4%) |
| IDH_mutation_status, n (%) | Mutant | 356 (55.5%) |
|  | Wildtype | 286 (44.5%) |
| 1p19q_codeletion_status, n (%) | Codel | 145 (23.3%) |
|  | Non-codel | 478 (76.7%) |

PRS: Primary, Recurrent, Secondary; A: Astrocytoma; AA: Anaplastic astrocytoma; AO: Anaplastic oligodendroglioma; AOA: anaplastic oligoastrocytoma; GBM: Glioblastoma multiforme; OA: oligoastrocytoma; rA: Recurrent astrocytoma; rAA: Recurrent anaplastic astrocytoma; rAO: Recurrent anaplastic oligodendroglioma; rAOA: Recurrent anaplastic oligoastrocytoma; rGBM: Recurrent glioblastoma multiforme; rO: Recurrent oligodendroglioma; rOA: Recurrent oligoastrocytoma; IDH: Isocitrate dehydrogenase

Supplement Table 3. Univariate regression and multivariate regression analysis of other genes in H2B family.

| Characteristics | Total(N) | HR(95% CI) (Uni) | P value | HR(95% CI) (Multi) | P value |
| --- | --- | --- | --- | --- | --- |
| WHO grade | 634 |  |  |  |  |
| G2 | 223 | Reference |  |  |  |
| G3 | 243 | 2.999 (2.007-4.480) | <0.001 | 2.228 (1.442-3.441) | <0.001 |
| G4 | 168 | 18.615 (12.460-27.812) | <0.001 | 4.976 (2.797-8.853) | <0.001 |
| 1p/19q codeletion | 688 |  |  |  |  |
| non-codel | 518 | Reference |  |  |  |
| codel | 170 | 0.226 (0.147-0.347) | <0.001 | 0.739 (0.411-1.330) | 0.313 |
| IDH status | 685 |  |  |  |  |
| WT | 246 | Reference |  |  |  |
| Mut | 439 | 0.117 (0.090-0.152) | <0.001 | 0.320 (0.207-0.495) | <0.001 |
| Age | 695 |  |  |  |  |
| <=60 | 552 | Reference |  |  |  |
| >60 | 143 | 4.668 (3.598-6.056) | <0.001 | 1.510 (1.104-2.064) | 0.010 |
| Gender | 695 |  |  |  |  |
| Female | 297 | Reference |  |  |  |
| Male | 398 | 1.262 (0.988-1.610) | 0.062 | 1.108 (0.837-1.467) | 0.474 |
| Race | 682 |  |  |  |  |
| Asian | 13 | Reference |  |  |  |
| Black or African American | 33 | 1.578 (0.453-5.494) | 0.474 |  |  |
| White | 636 | 1.176 (0.376-3.677) | 0.780 |  |  |
| H2BC3 | 695 | 1.671 (1.369-2.039) | <0.001 | 1.333 (0.738-2.408) | 0.341 |
| H2BC4 | 695 | 1.680 (1.499-1.883) | <0.001 | 1.229 (0.961-1.571) | 0.101 |
| H2BC6 | 695 | 1.794 (1.522-2.115) | <0.001 | 1.029 (0.737-1.438) | 0.865 |
| H2BC7 | 695 | 1.260 (1.101-1.443) | <0.001 | 0.892 (0.649-1.226) | 0.482 |
| H2BC8 | 695 | 1.466 (1.326-1.621) | <0.001 | 0.887 (0.697-1.129) | 0.329 |
| H2BC10 | 695 | 1.779 (1.552-2.039) | <0.001 | 0.880 (0.544-1.425) | 0.604 |
| H2BC12 | 695 | 1.722 (1.592-1.861) | <0.001 | 1.121 (0.894-1.406) | 0.323 |
| H2BC13 | 695 | 1.693 (1.421-2.018) | <0.001 | 0.874 (0.590-1.296) | 0.504 |
| H2BC14 | 695 | 2.416 (2.012-2.901) | <0.001 | 1.318 (0.823-2.110) | 0.250 |
| H2BC15 | 695 | 1.419 (1.145-1.757) | 0.001 | 0.877 (0.585-1.314) | 0.524 |
| H2BC17 | 695 | 1.635 (1.476-1.813) | <0.001 | 0.907 (0.712-1.154) | 0.426 |
| H2BC18 | 695 | 1.942 (1.617-2.331) | <0.001 | 0.982 (0.606-1.591) | 0.940 |

IDH: Isocitrate dehydrogenase; WT: Wild-type; Mut: Mutant. P<0.05 is considered statistically significant

Supplement Table 4. Correlation between H2BC5 and immune cell biomarkers.

| Cell Type | Marker | GBM | | | | LGG | | | |
| --- | --- | --- | --- | --- | --- | --- | --- | --- | --- |
|  |  | None | | purity | | None | | purity | |
|  |  | Cor | *P* | Cor | *P* | Cor | *P* | Cor | *P* |
| Neutrophils | FCGR3A | 0.163 | 0.157 | 0.202 | 0.117 | 0.193 | 0.000 | 0.199 | 0.001 |
|  | CEACAM8 | -0.014 | 0.925 | -0.063 | 0.682 | -0.018 | 0.814 | -0.015 | 0.870 |
|  | FUT4 | 0.137 | 0.247 | 0.172 | 0.198 | 0.163 | 0.003 | 0.171 | 0.005 |
| Eosinophils | CCR3 | 0.178 | 0.106 | 0.178 | 0.174 | 0.145 | 0.010 | 0.177 | 0.003 |
|  | SIGLEC8 | 0.066 | 0.617 | 0.056 | 0.725 | 0.076 | 0.283 | 0.077 | 0.204 |
| Basophils | FCER1A | -0.081 | 0.525 | -0.080 | 0.607 | 0.067 | 0.350 | 0.083 | 0.164 |
|  | KIT | -0.074 | 0.572 | -0.049 | 0.765 | -0.036 | 0.669 | -0.029 | 0.692 |
|  | ENPP3 | -0.202 | 0.053 | -0.200 | 0.093 | -0.177 | 0.002 | -0.162 | 0.002 |
| Mast cells | TPSAB1 | 0.099 | 0.409 | 0.125 | 0.350 | -0.003 | 0.981 | -0.004 | 0.956 |
| M MDSCs/PMN MDSCs | HLA-DRB1 | 0.098 | 0.413 | 0.090 | 0.559 | 0.200 | 0.000 | 0.195 | 0.000 |
|  | HLA-DRA | 0.083 | 0.515 | 0.111 | 0.423 | 0.238 | 0.000 | 0.230 | 0.000 |
| pDCs | IL3RA | -0.191 | 0.072 | -0.166 | 0.182 | -0.114 | 0.070 | -0.104 | 0.072 |
|  | CD83 | 0.124 | 0.277 | 0.182 | 0.134 | 0.111 | 0.052 | 0.133 | 0.029 |
| cDC1s | XCR1 | 0.127 | 0.139 | 0.142 | 0.002 | 0.127 | 0.139 | 0.142 | 0.002 |
|  | CLEC9A | -0.037 | 0.788 | -0.024 | 0.895 | -0.063 | 0.382 | -0.051 | 0.439 |
| cDC2s | CD1c | -0.024 | 0.882 | -0.022 | 0.916 | 0.117 | 0.045 | 0.123 | 0.073 |
|  | SIRPA | -0.012 | 0.939 | -0.006 | 0.981 | -0.066 | 0.350 | -0.071 | 0.250 |
| Macrophages | CD68 | 0.188 | 0.094 | 0.204 | 0.113 | 0.162 | 0.003 | 0.164 | 0.007 |
| M1-like | CD86 | 0.153 | 0.182 | 0.181 | 0.166 | 0.164 | 0.003 | 0.183 | 0.002 |
|  | CD80 | 0.037 | 0.797 | 0.041 | 0.834 | 0.207 | 0.000 | 0.210 | 0.000 |
|  | NOS2 | -0.125 | 0.299 | -0.140 | 0.317 | -0.113 | 0.058 | -0.102 | 0.138 |
| M2-like | CD163 | 0.221 | 0.032 | 0.238 | 0.036 | 0.248 | 0.000 | 0.244 | 0.000 |
|  | MRC1 | 0.125 | 0.300 | 0.103 | 0.510 | -0.048 | 0.503 | -0.057 | 0.484 |
| Monocytes | CD14 | 0.161 | 0.160 | 0.195 | 0.130 | 0.128 | 0.025 | 0.134 | 0.036 |
| T cells | CD247 | 0.098 | 0.413 | 0.121 | 0.365 | 0.173 | 0.001 | 0.201 | 0.000 |
|  | PTPRC | 0.121 | 0.296 | 0.152 | 0.235 | 0.218 | 0.000 | 0.201 | 0.000 |
|  | SELL | 0.196 | 0.063 | 0.222 | 0.054 | -0.104 | 0.104 | -0.100 | 0.083 |
|  | CCR7 | 0.095 | 0.434 | 0.127 | 0.345 | 0.182 | 0.000 | 0.186 | 0.001 |
|  | HNF1A | 0.047 | 0.733 | 0.033 | 0.847 | 0.112 | 0.078 | 0.086 | 0.145 |
|  | TOX2 | 0.038 | 0.643 | 0.002 | 0.984 | -0.007 | 0.882 | -0.008 | 0.859 |
|  | TIGIT | 0.107 | 0.462 | 0.127 | 0.443 | -0.042 | 0.678 | -0.027 | 0.755 |
|  | HAVCR2 | 0.087 | 0.573 | 0.117 | 0.481 | 0.153 | 0.016 | 0.138 | 0.016 |
| Cytotoxic T Cells | CD8A | 0.082 | 0.522 | 0.128 | 0.347 | 0.128 | 0.026 | 0.156 | 0.011 |
| Helper T Cells | CD4 | 0.142 | 0.262 | 0.172 | 0.240 | 0.131 | 0.047 | 0.128 | 0.030 |
| Th1 | TBX21 | -0.032 | 0.826 | -0.010 | 0.959 | 0.217 | 0.000 | 0.222 | 0.000 |
|  | IFNG | 0.107 | 0.462 | 0.092 | 0.617 | 0.132 | 0.046 | 0.119 | 0.046 |
| Th2 | GATA-3 | 0.126 | 0.285 | 0.182 | 0.166 | 0.208 | 0.000 | 0.208 | 0.000 |
|  | IL4 | -0.024 | 0.900 | -0.020 | 0.945 | 0.024 | 0.822 | 0.024 | 0.774 |
| Th17 | RORC | **0.355** | **0.000** | **0.328** | **0.003** | 0.156 | 0.014 | 0.163 | 0.003 |
|  | IL17A | 0.074 | 0.637 | 0.081 | 0.671 | 0.050 | 0.615 | 0.042 | 0.604 |
| Th9 | PDCD1 | 0.147 | 0.201 | 0.182 | 0.166 | 0.191 | 0.000 | 0.191 | 0.002 |
|  | IL-9 | -0.165 | 0.166 | -0.151 | 0.321 | -0.202 | 0.001 | -0.181 | 0.001 |
| Tfh | BCL-6 | 0.051 | 0.715 | 0.080 | 0.600 | 0.058 | 0.382 | 0.045 | 0.576 |
|  | CXCR5 | 0.150 | 0.183 | 0.196 | 0.126 | 0.108 | 0.072 | 0.114 | 0.086 |
|  | IL21 | 0.083 | 0.590 | 0.017 | 0.959 | 0.051 | 0.607 | 0.029 | 0.735 |
| Th22 | IL22 | -0.059 | 0.712 | -0.061 | 0.755 | 0.000 | 0.996 | -0.001 | 0.989 |
|  | AHR | 0.033 | 0.827 | 0.031 | 0.875 | 0.062 | 0.355 | 0.071 | 0.347 |
| Treg | FOXP3 | 0.247 | 0.018 | 0.254 | 0.032 | 0.090 | 0.148 | 0.102 | 0.140 |
|  | IL2RA | 0.120 | 0.319 | 0.155 | 0.256 | 0.180 | 0.001 | 0.186 | 0.002 |
| NKT Cells | NCAM1 | -0.026 | 0.888 | -0.018 | 0.956 | -0.002 | 0.991 | 0.007 | 0.965 |
| NK Cells | CD69 | -0.056 | 0.723 | -0.030 | 0.895 | 0.229 | 0.000 | 0.217 | 0.000 |
| PB Cytotoxic NK Cells | GZMA | 0.125 | 0.353 | 0.160 | 0.290 | 0.200 | 0.001 | 0.205 | 0.000 |
|  | GZMB | -0.009 | 0.975 | -0.004 | 0.991 | 0.148 | 0.022 | 0.139 | 0.015 |
|  | GZMH | 0.035 | 0.830 | 0.065 | 0.735 | 0.142 | 0.032 | 0.132 | 0.024 |
|  | GZMK | 0.123 | 0.362 | 0.155 | 0.309 | 0.196 | 0.001 | 0.197 | 0.000 |
|  | GZMM | 0.062 | 0.698 | 0.090 | 0.625 | -0.004 | 0.991 | 0.005 | 0.975 |
|  | PRF1 | 0.024 | 0.900 | 0.063 | 0.750 | 0.193 | 0.001 | 0.179 | 0.001 |
| PB Immature/Regulatory NK Cells | NCR1 | 0.041 | 0.793 | 0.069 | 0.718 | 0.071 | 0.410 | 0.060 | 0.440 |
|  | NCR2 | 0.004 | 0.985 | 0.021 | 0.939 | 0.034 | 0.750 | 0.043 | 0.597 |
|  | NCR3 | -0.026 | 0.846 | -0.011 | 0.965 | 0.134 | 0.030 | 0.128 | 0.019 |
| B cells | CD19 | -0.147 | 0.190 | -0.080 | 0.600 | 0.021 | 0.794 | 0.010 | 0.923 |
|  | CD27 | 0.163 | 0.127 | 0.189 | 0.115 | 0.134 | 0.030 | 0.134 | 0.014 |
|  | CD38 | 0.027 | 0.852 | 0.059 | 0.713 | -0.098 | 0.103 | -0.118 | 0.073 |
| Plasma Cells | TNFRSF17 | -0.075 | 0.556 | -0.054 | 0.775 | -0.013 | 0.903 | -0.016 | 0.827 |
|  | SDC1 | 0.170 | 0.115 | 0.167 | 0.174 | 0.245 | 0.000 | 0.226 | 0.000 |

MDSDs: Myeloid-Derived Suppressor Cells; M MDSCs/PMN MDSCs: Monocytic MDSCs/Polymorphonuclear MDSCs; cDCs: Conventional Dendritic Cells; Th: Helper T Cells; Cor: Correlation coefficient; *P*: Adjusted p value; P<0.05 is considered statistically significant

Supplement Table 5. Correlation between H2BC9 and immune cell biomarkers.

| Cell Type | Marker | GBM | | | | LGG | | | |
| --- | --- | --- | --- | --- | --- | --- | --- | --- | --- |
|  |  | None | | purity | | None | | purity | |
|  |  | Cor | P | Cor | P | Cor | P | Cor | P |
| Neutrophils | FCGR3A | -0.046 | 0.763 | 0.141 | 0.283 | **0.397** | **0.000** | **0.399** | **0.000** |
|  | CEACAM8 | -0.063 | 0.646 | -0.091 | 0.535 | -0.015 | 0.857 | -0.009 | 0.921 |
|  | FUT4 | -0.013 | 0.952 | -0.009 | 0.967 | **0.310** | **0.000** | **0.302** | **0.000** |
| Eosinophils | CCR3 | -0.013 | 0.943 | 0.016 | 0.964 | 0.260 | 0.000 | 0.253 | 0.000 |
|  | SIGLEC8 | -0.085 | 0.512 | 0.004 | 0.982 | 0.163 | 0.002 | 0.165 | 0.004 |
| Basophils | FCER1A | -0.051 | 0.719 | 0.072 | 0.636 | 0.119 | 0.035 | 0.107 | 0.096 |
|  | KIT | -0.053 | 0.713 | -0.114 | 0.416 | -0.166 | 0.002 | -0.159 | 0.006 |
|  | ENPP3 | -0.154 | 0.170 | -0.189 | 0.121 | 0.004 | 0.959 | 0.001 | 0.989 |
| Mast cells | TPSAB1 | 0.110 | 0.385 | 0.147 | 0.261 | 0.005 | 0.947 | 0.017 | 0.852 |
| M MDSCs/PMN MDSCs | HLA-DRB1 | -0.032 | 0.830 | 0.098 | 0.496 | **0.366** | **0.000** | **0.365** | **0.000** |
|  | HLA-DRA | -0.071 | 0.592 | 0.072 | 0.636 | **0.374** | **0.000** | **0.376** | **0.000** |
| pDCs | IL3RA | 0.013 | 0.930 | 0.087 | 0.556 | **0.055** | **0.430** | **0.035** | **0.675** |
|  | CD83 | -0.024 | 0.878 | 0.026 | 0.881 | 0.129 | 0.020 | 0.131 | 0.030 |
| cDC1s | XCR1 | 0.014 | 0.862 | 0.194 | 0.000 | 0.058 | 0.498 | 0.185 | 0.000 |
|  | CLEC9A | **-0.359** | **0.000** | **-0.318** | **0.002** | -0.052 | 0.462 | -0.075 | 0.294 |
| cDC2s | CD1c | 0.003 | 0.990 | 0.074 | 0.605 | 0.156 | 0.003 | 0.131 | 0.029 |
|  | SIRPA | -0.255 | 0.010 | -0.229 | 0.049 | -0.081 | 0.190 | -0.080 | 0.244 |
| Macrophages | CD68 | -0.010 | 0.973 | 0.202 | 0.091 | **0.326** | **0.000** | **0.327** | **0.000** |
| M1-like | CD86 | -0.090 | 0.499 | 0.051 | 0.765 | **0.313** | **0.000** | **0.316** | **0.000** |
|  | CD80 | 0.081 | 0.560 | 0.096 | 0.515 | **0.372** | **0.000** | **0.372** | **0.000** |
|  | NOS2 | 0.161 | 0.160 | 0.133 | 0.318 | 0.040 | 0.609 | 0.043 | 0.593 |
| M2-like | CD163 | 0.075 | 0.570 | 0.256 | 0.021 | **0.300** | **0.000** | 0.280 | 0.000 |
|  | MRC1 | -0.026 | 0.874 | 0.135 | 0.312 | -0.137 | 0.012 | -0.165 | 0.003 |
| Monocytes | CD14 | 0.037 | 0.653 | 0.293 | 0.000 | 0.261 | 0.002 | 0.28 | 0.000 |
| T cells | CD247 | 0.191 | 0.070 | **0.300** | **0.005** | **0.372** | **0.000** | **0.396** | **0.000** |
|  | PTPRC | -0.043 | 0.766 | 0.084 | 0.567 | **0.341** | **0.000** | **0.347** | **0.000** |
|  | SELL | -0.085 | 0.512 | 0.040 | 0.804 | -0.252 | 0.000 | -0.267 | 0.000 |
|  | CCR7 | 0.245 | 0.015 | 0.375 | 0.000 | **0.305** | **0.000** | **0.305** | **0.000** |
|  | HNF1A | 0.269 | 0.006 | 0.165 | 0.198 | -0.032 | 0.658 | -0.025 | 0.777 |
|  | TOX2 | 0.091 | 0.266 | 0.141 | 0.101 | -0.207 | 0.000 | -0.209 | 0.000 |
|  | TIGIT | 0.060 | 0.685 | 0.154 | 0.268 | 0.061 | 0.381 | 0.051 | 0.534 |
|  | HAVCR2 | -0.085 | 0.537 | 0.072 | 0.645 | **0.348** | **0.000** | **0.357** | **0.000** |
| Cytotoxic T Cells | CD8A | 0.215 | 0.039 | 0.299 | 0.004 | 0.213 | 0.000 | 0.226 | 0.000 |
| Helper T Cells | CD4 | 0.001 | 0.987 | 0.184 | 0.154 | **0.309** | **0.000** | **0.301** | **0.000** |
| Th1 | TBX21 | **0.324** | **0.001** | 0.262 | 0.017 | 0.289 | 0.000 | 0.283 | 0.000 |
|  | IFNG | 0.089 | 0.520 | 0.085 | 0.578 | 0.229 | 0.000 | 0.238 | 0.000 |
| Th2 | GATA-3 | 0.035 | 0.831 | 0.021 | 0.943 | **0.342** | **0.000** | **0.337** | **0.000** |
|  | IL4 | -0.022 | 0.912 | -0.038 | 0.845 | 0.074 | 0.273 | 0.081 | 0.274 |
| Th17 | RORC | 0.088 | 0.520 | 0.164 | 0.229 | 0.093 | 0.138 | 0.106 | 0.117 |
|  | IL17A | -0.008 | 0.960 | 0.028 | 0.885 | 0.025 | 0.774 | 0.019 | 0.850 |
| Th9 | PDCD1 | 0.227 | 0.026 | 0.276 | 0.010 | **0.364** | **0.000** | **0.355** | **0.000** |
|  | IL-9 | 0.012 | 0.941 | 0.074 | 0.633 | -0.159 | 0.003 | -0.156 | 0.008 |
| Tfh | BCL-6 | -0.164 | 0.137 | -0.187 | 0.129 | -0.011 | 0.903 | -0.015 | 0.908 |
|  | CXCR5 | 0.054 | 0.723 | 0.112 | 0.417 | 0.237 | 0.000 | 0.226 | 0.000 |
|  | IL21 | -0.037 | 0.825 | -0.036 | 0.849 | 0.110 | 0.064 | 0.094 | 0.179 |
| Th22 | IL22 | 0.055 | 0.718 | -0.004 | 0.990 | 0.055 | 0.452 | 0.048 | 0.554 |
|  | AHR | 0.189 | 0.081 | 0.270 | 0.013 | 0.261 | 0.000 | 0.263 | 0.000 |
| Treg | FOXP3 | -0.019 | 0.922 | 0.016 | 0.929 | -0.031 | 0.692 | -0.021 | 0.817 |
|  | IL2RA | 0.050 | 0.738 | 0.196 | 0.104 | 0.209 | 0.000 | 0.186 | 0.001 |
| NKT Cells | NCAM1 | 0.055 | 0.723 | -0.059 | 0.719 | **-0.320** | **0.000** | **-0.343** | **0.000** |
| NK Cells | CD69 | -0.083 | 0.551 | 0.015 | 0.942 | **0.378** | **0.000** | **0.385** | **0.000** |
| PB Cytotoxic NK Cells | GZMA | -0.044 | 0.781 | 0.036 | 0.849 | **0.347** | **0.000** | **0.352** | **0.000** |
|  | GZMB | 0.004 | 0.976 | 0.077 | 0.619 | 0.228 | 0.000 | 0.225 | 0.000 |
|  | GZMH | 0.089 | 0.520 | 0.153 | 0.274 | **0.321** | **0.000** | **0.320** | **0.000** |
|  | GZMK | 0.015 | 0.932 | 0.102 | 0.515 | **0.346** | **0.000** | **0.361** | **0.000** |
|  | GZMM | 0.028 | 0.875 | 0.057 | 0.727 | 0.176 | 0.001 | 0.169 | 0.003 |
|  | PRF1 | 0.159 | 0.183 | 0.311 | 0.003 | 0.303 | 0.000 | 0.296 | 0.000 |
| PB Immature/Regulatory NK Cells | NCR1 | 0.091 | 0.507 | 0.101 | 0.518 | 0.163 | 0.002 | 0.146 | 0.015 |
|  | NCR2 | -0.051 | 0.754 | -0.116 | 0.438 | -0.009 | 0.927 | -0.014 | 0.886 |
|  | NCR3 | -0.047 | 0.730 | 0.001 | 0.992 | 0.094 | 0.112 | 0.100 | 0.119 |
| B cells | CD19 | -0.055 | 0.721 | 0.040 | 0.849 | **0.325** | **0.000** | **0.320** | **0.000** |
|  | CD27 | 0.000 | 0.996 | 0.101 | 0.436 | 0.196 | 0.000 | 0.201 | 0.000 |
|  | CD38 | -0.044 | 0.785 | -0.009 | 0.974 | -0.032 | 0.702 | -0.041 | 0.636 |
| Plasma Cells | TNFRSF17 | 0.092 | 0.426 | 0.086 | 0.526 | 0.106 | 0.065 | 0.102 | 0.119 |
|  | SDC1 | 0.230 | 0.024 | 0.247 | 0.034 | **0.353** | **0.000** | **0.348** | **0.000** |

MDSDs: Myeloid-Derived Suppressor Cells; M MDSCs/PMN MDSCs: Monocytic MDSCs/Polymorphonuclear MDSCs; cDCs: Conventional Dendritic Cells; Th: Helper T Cells; Cor: Correlation coefficient; *P*: Adjusted p value; P< 0.05 is considered statistically significant

Supplement Table 6. Correlation between H2BC11 and immune cell biomarkers.

| Cell Type | Marker | GBM | | | | LGG | | | |
| --- | --- | --- | --- | --- | --- | --- | --- | --- | --- |
|  |  | None | | purity | | None | | purity | |
|  |  | Cor | *P* | Cor | *P* | Cor | *P* | Cor | *P* |
| Neutrophils | FCGR3A | 0.214 | 0.035 | 0.251 | 0.018 | **0.406** | **0.000** | **0.383** | **0.000** |
|  | CEACAM8 | 0.058 | 0.706 | 0.029 | 0.870 | 0.025 | 0.773 | 0.020 | 0.823 |
|  | FUT4 | 0.073 | 0.571 | 0.101 | 0.410 | **0.439** | **0.000** | **0.401** | **0.000** |
| Eosinophils | CCR3 | 0.183 | 0.088 | 0.165 | 0.160 | 0.292 | 0.000 | 0.279 | 0.000 |
|  | SIGLEC8 | 0.173 | 0.109 | 0.163 | 0.163 | 0.263 | 0.000 | 0.210 | 0.000 |
| Basophils | FCER1A | -0.025 | 0.883 | -0.011 | 0.939 | 0.146 | 0.007 | 0.128 | 0.028 |
|  | KIT | -0.018 | 0.914 | -0.003 | 0.982 | -0.145 | 0.007 | -0.133 | 0.020 |
|  | ENPP3 | -0.231 | 0.022 | -0.229 | 0.036 | -0.063 | 0.355 | -0.095 | 0.120 |
| Mast cells | TPSAB1 | 0.129 | 0.284 | 0.141 | 0.243 | -0.062 | 0.356 | -0.030 | 0.699 |
| M MDSCs/ PMN MDSCs | HLA-DRB1 | 0.151 | 0.186 | 0.154 | 0.194 | **0.357** | **0.000** | **0.327** | **0.000** |
|  | HLA-DRA | 0.197 | 0.062 | 0.218 | 0.047 | **0.389** | **0.000** | **0.357** | **0.000** |
| pDCs | IL3RA | -0.160 | 0.150 | -0.137 | 0.257 | -0.006 | 0.956 | -0.037 | 0.621 |
|  | CD83 | 0.087 | 0.515 | 0.132 | 0.279 | 0.245 | 0.000 | 0.208 | 0.000 |
| cDC1s | XCR1 | 0.130 | 0.108 | 0.125 | 0.004 | 0.120 | 0.163 | 0.122 | 0.008 |
|  | CLEC9A | -0.140 | 0.229 | -0.119 | 0.341 | 0.102 | 0.079 | 0.033 | 0.662 |
| cDC2s | CD1c | 0.016 | 0.938 | -0.003 | 0.976 | 0.160 | 0.003 | 0.153 | 0.008 |
|  | SIRPA | -0.179 | 0.098 | -0.161 | 0.169 | -0.118 | 0.035 | -0.169 | 0.002 |
| Macrophages | CD68 | 0.245 | 0.013 | 0.274 | 0.008 | **0.348** | **0.000** | **0.304** | **0.000** |
| M1-like | CD86 | 0.210 | 0.040 | 0.234 | 0.029 | **0.375** | **0.000** | **0.332** | **0.000** |
|  | CD80 | 0.122 | 0.298 | 0.128 | 0.287 | **0.309** | **0.000** | 0.290 | 0.000 |
|  | NOS2 | -0.134 | 0.240 | -0.146 | 0.213 | -0.026 | 0.741 | -0.040 | 0.561 |
| M2-like | CD163 | 0.240 | 0.016 | 0.238 | 0.027 | 0.278 | 0.000 | 0.261 | 0.000 |
|  | MRC1 | 0.201 | 0.051 | 0.180 | 0.114 | -0.063 | 0.321 | -0.099 | 0.105 |
| Monocytes | CD14 | 0.178 | 0.028 | 0.198 | 0.020 | 0.289 | 0.000 | 0.258 | 0.000 |
| T cells | CD247 | 0.175 | 0.106 | 0.199 | 0.074 | 0.293 | 0.000 | 0.277 | 0.000 |
|  | PTPRC | 0.151 | 0.188 | 0.165 | 0.160 | 0.406 | 0.000 | 0.362 | 0.000 |
|  | SELL | 0.220 | 0.031 | 0.235 | 0.029 | -0.209 | 0.000 | -0.225 | 0.000 |
|  | CCR7 | 0.229 | 0.023 | 0.255 | 0.017 | 0.218 | 0.000 | 0.213 | 0.000 |
|  | HNF1A | 0.107 | 0.399 | 0.112 | 0.376 | 0.003 | 0.968 | 0.049 | 0.477 |
|  | TOX2 | 0.080 | 0.323 | 0.080 | 0.351 | -0.087 | 0.048 | -0.074 | 0.107 |
|  | TIGIT | 0.143 | 0.274 | 0.155 | 0.241 | -0.054 | 0.517 | -0.086 | 0.218 |
|  | HAVCR2 | 0.194 | 0.098 | 0.230 | 0.053 | **0.402** | **0.000** | **0.359** | **0.000** |
| Cytotoxic T Cells | CD8A | 0.175 | 0.107 | 0.218 | 0.051 | 0.186 | 0.000 | 0.144 | 0.011 |
| Helper T Cells | CD4 | 0.198 | 0.089 | 0.225 | 0.060 | 0.298 | 0.000 | 0.249 | 0.000 |
| Th1 | TBX21 | 0.166 | 0.135 | 0.179 | 0.121 | 0.268 | 0.000 | 0.268 | 0.000 |
|  | IFNG | 0.137 | 0.310 | 0.105 | 0.482 | 0.174 | 0.001 | 0.162 | 0.005 |
| Th2 | GATA-3 | 0.008 | 0.981 | 0.040 | 0.797 | 0.323 | 0.000 | 0.297 | 0.000 |
|  | IL4 | -0.089 | 0.579 | -0.088 | 0.580 | 0.034 | 0.737 | 0.039 | 0.673 |
| Th17 | RORC | 0.318 | 0.001 | 0.316 | 0.003 | -0.004 | 0.985 | -0.014 | 0.907 |
|  | IL17A | -0.035 | 0.870 | -0.036 | 0.872 | -0.017 | 0.885 | -0.021 | 0.854 |
| Th9 | PDCD1 | 0.266 | 0.006 | 0.281 | 0.006 | 0.265 | 0.000 | 0.241 | 0.000 |
|  | IL-9 | -0.149 | 0.254 | -0.141 | 0.301 | -0.134 | 0.020 | -0.152 | 0.009 |
| Tfh | BCL-6 | -0.064 | 0.649 | -0.052 | 0.723 | 0.074 | 0.239 | 0.076 | 0.237 |
|  | CXCR5 | 0.150 | 0.185 | 0.179 | 0.121 | 0.148 | 0.006 | 0.140 | 0.014 |
|  | IL21 | 0.053 | 0.777 | 0.020 | 0.926 | 0.136 | 0.017 | 0.127 | 0.045 |
| Th22 | IL22 | -0.035 | 0.870 | -0.039 | 0.856 | 0.056 | 0.495 | 0.060 | 0.445 |
|  | AHR | 0.138 | 0.221 | 0.140 | 0.235 | 0.270 | 0.000 | 0.229 | 0.000 |
| Treg | FOXP3 | 0.272 | 0.005 | 0.272 | 0.009 | -0.036 | 0.613 | -0.008 | 0.921 |
|  | IL2RA | 0.151 | 0.169 | 0.157 | 0.180 | 0.164 | 0.002 | 0.167 | 0.003 |
| NKT Cells | NCAM1 | 0.004 | 0.992 | 0.026 | 0.907 | -0.219 | 0.000 | -0.186 | 0.001 |
| NK Cells | CD69 | 0.060 | 0.746 | 0.089 | 0.569 | 0.388 | 0.000 | 0.354 | 0.000 |
| PB Cytotoxic NK Cells | GZMA | 0.187 | 0.114 | 0.210 | 0.083 | 0.247 | 0.000 | 0.231 | 0.000 |
|  | GZMB | 0.001 | 0.997 | -0.029 | 0.907 | 0.131 | 0.023 | 0.153 | 0.009 |
|  | GZMK | 0.120 | 0.411 | 0.131 | 0.340 | 0.191 | 0.000 | 0.175 | 0.002 |
|  | GZMM | 0.176 | 0.147 | 0.199 | 0.103 | 0.264 | 0.000 | 0.243 | 0.000 |
|  | GZMH | 0.072 | 0.688 | 0.074 | 0.673 | 0.063 | 0.429 | 0.062 | 0.410 |
|  | PRF1 | 0.095 | 0.541 | 0.119 | 0.400 | 0.251 | 0.000 | 0.240 | 0.000 |
| PB Immature/Regulatory NK Cells | NCR1 | 0.056 | 0.766 | 0.069 | 0.703 | 0.137 | 0.016 | 0.150 | 0.011 |
|  | NCR2 | 0.010 | 0.969 | -0.007 | 0.989 | 0.020 | 0.861 | 0.010 | 0.937 |
|  | NCR3 | 0.057 | 0.770 | 0.088 | 0.552 | 0.050 | 0.518 | 0.066 | 0.368 |
| B cells | CD19 | -0.056 | 0.702 | -0.001 | 0.995 | 0.217 | 0.000 | 0.183 | 0.001 |
|  | CD27 | 0.214 | 0.055 | 0.223 | 0.056 | 0.136 | 0.019 | 0.151 | 0.008 |
|  | CD38 | 0.128 | 0.274 | 0.148 | 0.211 | 0.034 | 0.659 | 0.001 | 0.992 |
| Plasma Cells | TNFRSF17 | -0.040 | 0.864 | -0.048 | 0.794 | 0.014 | 0.924 | 0.013 | 0.893 |
|  | SDC1 | 0.182 | 0.117 | 0.164 | 0.202 | 0.284 | 0.000 | 0.314 | 0.000 |

MDSDs: Myeloid-Derived Suppressor Cells; M MDSCs/PMN MDSCs: Monocytic MDSCs/Polymorphonuclear MDSCs; cDCs: Conventional Dendritic Cells; Th: Helper T Cells; Cor: Correlation coefficient; *P*: Adjusted p value; P<0.05 is considered statistically significant

Supplement Table 7. Correlation between H2BC21 and immune cell biomarkers.

| Cell Type | Marker | GBM | | | | LGG | | | |
| --- | --- | --- | --- | --- | --- | --- | --- | --- | --- |
|  |  | None | | purity | | None | | purity | |
|  |  | Cor | *P* | Cor | *P* | Cor | *P* | Cor | *P* |
| Neutrophils | FCGR3A | 0.102 | 0.393 | 0.154 | 0.241 | 0.118 | 0.043 | 0.106 | 0.107 |
|  | CEACAM8 | 0.056 | 0.672 | 0.048 | 0.784 | 0.030 | 0.672 | 0.031 | 0.725 |
|  | FUT4 | 0.077 | 0.540 | 0.123 | 0.393 | 0.218 | 0.000 | 0.191 | 0.001 |
| Eosinophils | CCR3 | 0.115 | 0.306 | 0.189 | 0.107 | 0.066 | 0.273 | 0.070 | 0.319 |
|  | SIGLEC8 | 0.155 | 0.166 | 0.185 | 0.143 | 0.133 | 0.017 | 0.069 | 0.387 |
| Basophils | FCER1A | -0.030 | 0.855 | -0.054 | 0.748 | 0.011 | 0.903 | 0.004 | 0.971 |
|  | KIT | 0.251 | 0.013 | **0.312** | **0.004** | -0.049 | 0.482 | -0.051 | 0.541 |
|  | ENPP3 | -0.091 | 0.472 | -0.103 | 0.514 | -0.094 | 0.113 | -0.123 | 0.051 |
| Mast cells | TPSAB1 | -0.012 | 0.939 | 0.013 | 0.953 | -0.094 | 0.113 | -0.078 | 0.290 |
| M MDSCs/ PMN MDSCs | HLA-DRB1 | 0.060 | 0.650 | 0.080 | 0.626 | 0.136 | 0.014 | 0.124 | 0.050 |
|  | HLA-DRA | -0.010 | 0.945 | 0.034 | 0.865 | 0.171 | 0.002 | 0.157 | 0.008 |
| pDCs | IL3RA | -0.123 | 0.302 | -0.091 | 0.574 | -0.106 | 0.067 | -0.124 | 0.050 |
|  | CD83 | 0.111 | 0.363 | 0.184 | 0.144 | 0.112 | 0.052 | 0.093 | 0.174 |
| cDC1s | XCR1 | -0.010 | 0.904 | 0.061 | 0.166 | 0.012 | 0.885 | 0.065 | 0.154 |
|  | CLEC9A | 0.132 | 0.258 | 0.112 | 0.476 | 0.055 | 0.413 | -0.013 | 0.906 |
| cDC2s | CD1c | -0.115 | 0.352 | -0.095 | 0.530 | 0.059 | 0.373 | 0.072 | 0.305 |
|  | SIRPA | 0.131 | 0.262 | 0.147 | 0.291 | 0.066 | 0.310 | 0.021 | 0.828 |
| Macrophages | CD68 | 0.140 | 0.224 | 0.193 | 0.118 | 0.121 | 0.036 | 0.089 | 0.194 |
| M1-like | CD86 | 0.111 | 0.354 | 0.176 | 0.165 | 0.168 | 0.002 | 0.140 | 0.023 |
|  | CD80 | 0.024 | 0.857 | 0.057 | 0.747 | 0.117 | 0.045 | 0.110 | 0.090 |
|  | NOS2 | -0.145 | 0.206 | -0.141 | 0.309 | -0.085 | 0.163 | -0.097 | 0.150 |
| M2-like | CD163 | 0.078 | 0.555 | 0.082 | 0.614 | 0.090 | 0.138 | 0.089 | 0.205 |
|  | MRC1 | 0.060 | 0.637 | 0.026 | 0.896 | -0.064 | 0.333 | -0.090 | 0.187 |
| Monocytes | CD14 | 0.069 | 0.394 | 0.095 | 0.272 | 0.056 | 0.202 | 0.043 | 0.351 |
| T cells | CD247 | 0.027 | 0.873 | 0.081 | 0.621 | 0.106 | 0.066 | 0.108 | 0.094 |
|  | PTPRC | 0.100 | 0.417 | 0.164 | 0.211 | 0.188 | 0.000 | 0.166 | 0.005 |
|  | SELL | 0.199 | 0.060 | 0.255 | 0.025 | -0.001 | 0.984 | -0.027 | 0.756 |
|  | CCR7 | 0.072 | 0.584 | 0.111 | 0.477 | 0.155 | 0.005 | 0.160 | 0.006 |
|  | HNF1A | 0.145 | 0.202 | 0.185 | 0.144 | 0.002 | 0.973 | 0.036 | 0.678 |
|  | TOX2 | 0.077 | 0.347 | -0.045 | 0.602 | -0.026 | 0.549 | -0.028 | 0.538 |
|  | TIGIT | 0.022 | 0.897 | 0.042 | 0.837 | 0.040 | 0.586 | 0.018 | 0.869 |
|  | HAVCR2 | 0.101 | 0.438 | 0.171 | 0.230 | 0.167 | 0.004 | 0.138 | 0.036 |
| Cytotoxic T Cells | CD8A | -0.015 | 0.900 | 0.047 | 0.758 | 0.102 | 0.074 | 0.075 | 0.283 |
| Helper T Cells | CD4 | 0.109 | 0.405 | 0.163 | 0.262 | 0.075 | 0.253 | 0.046 | 0.611 |
| Th1 | TBX21 | 0.033 | 0.794 | 0.088 | 0.536 | 0.167 | 0.002 | 0.190 | 0.001 |
|  | IFNG | 0.066 | 0.631 | 0.099 | 0.560 | 0.120 | 0.046 | 0.118 | 0.089 |
| Th2 | GATA-3 | 0.032 | 0.798 | 0.068 | 0.644 | 0.149 | 0.006 | 0.128 | 0.033 |
|  | IL4 | -0.090 | 0.498 | -0.102 | 0.550 | -0.021 | 0.817 | -0.010 | 0.927 |
| Th17 | RORC | 0.290 | 0.006 | **0.308** | **0.007** | 0.091 | 0.143 | 0.082 | 0.294 |
|  | IL17A | 0.093 | 0.486 | 0.105 | 0.523 | -0.032 | 0.667 | -0.034 | 0.725 |
| Th9 | PDCD1 | 0.141 | 0.222 | 0.207 | 0.085 | 0.095 | 0.119 | 0.084 | 0.232 |
|  | IL-9 | -0.059 | 0.669 | -0.140 | 0.375 | -0.089 | 0.155 | -0.114 | 0.107 |
| Tfh | BCL-6 | 0.144 | 0.185 | 0.173 | 0.156 | 0.090 | 0.121 | 0.088 | 0.185 |
|  | CXCR5 | 0.080 | 0.503 | 0.124 | 0.347 | 0.055 | 0.375 | 0.057 | 0.437 |
|  | IL21 | -0.022 | 0.897 | -0.073 | 0.679 | -0.008 | 0.930 | 0.018 | 0.873 |
| Th22 | IL22 | -0.075 | 0.581 | -0.083 | 0.621 | -0.069 | 0.307 | -0.060 | 0.493 |
|  | AHR | -0.008 | 0.959 | 0.002 | 0.995 | 0.054 | 0.405 | 0.041 | 0.669 |
| Treg | FOXP3 | 0.144 | 0.209 | 0.182 | 0.146 | 0.082 | 0.182 | 0.090 | 0.185 |
|  | IL2RA | 0.022 | 0.871 | 0.019 | 0.927 | 0.026 | 0.719 | 0.043 | 0.654 |
| NKT Cells | NCAM1 | 0.252 | 0.019 | 0.302 | 0.009 | 0.011 | 0.908 | 0.045 | 0.621 |
| NK Cells | CD69 | -0.048 | 0.746 | -0.008 | 0.965 | 0.201 | 0.000 | 0.187 | 0.002 |
| PB Cytotoxic NK Cells | GZMA | -0.023 | 0.897 | 0.021 | 0.925 | 0.092 | 0.140 | 0.087 | 0.266 |
|  | GZMB | -0.092 | 0.492 | -0.054 | 0.779 | 0.035 | 0.631 | 0.052 | 0.560 |
|  | GZMK | -0.062 | 0.656 | -0.007 | 0.970 | 0.053 | 0.462 | 0.058 | 0.502 |
|  | GZMM | -0.037 | 0.826 | 0.004 | 0.982 | 0.110 | 0.069 | 0.102 | 0.170 |
|  | GZMH | -0.091 | 0.495 | -0.045 | 0.821 | -0.037 | 0.612 | -0.052 | 0.560 |
|  | PRF1 | -0.023 | 0.897 | 0.007 | 0.970 | 0.080 | 0.218 | 0.098 | 0.192 |
| PB Immature/Regulatory NK Cells | NCR1 | -0.008 | 0.959 | 0.016 | 0.932 | 0.049 | 0.498 | 0.068 | 0.414 |
|  | NCR2 | -0.123 | 0.326 | -0.095 | 0.567 | 0.001 | 0.987 | -0.019 | 0.860 |
|  | NCR3 | 0.033 | 0.794 | 0.058 | 0.727 | 0.134 | 0.019 | 0.146 | 0.020 |
| B cells | CD19 | -0.025 | 0.837 | -0.009 | 0.969 | 0.011 | 0.871 | -0.014 | 0.872 |
|  | CD27 | -0.024 | 0.855 | 0.015 | 0.935 | 0.055 | 0.410 | 0.072 | 0.391 |
|  | CD38 | 0.284 | 0.004 | **0.346** | **0.001** | 0.072 | 0.222 | 0.030 | 0.707 |
| Plasma Cells | TNFRSF17 | 0.074 | 0.541 | 0.106 | 0.511 | -0.035 | 0.614 | -0.045 | 0.606 |
|  | SDC1 | 0.092 | 0.466 | 0.121 | 0.420 | 0.080 | 0.203 | 0.131 | 0.042 |

MDSDs: Myeloid-Derived Suppressor Cells; M MDSCs/PMN MDSCs: Monocytic MDSCs/Polymorphonuclear MDSCs; cDCs: Conventional Dendritic Cells; Th: Helper T Cells; Cor: Correlation coefficient; *P*: Adjusted p value; P< 0.05 is considered statistically significant.

Supplement Table 8. Enrichment analysis results of String database.

| Term ID | observed gene count | background gene count | strength | FDR |
| --- | --- | --- | --- | --- |
| hsa05034 | 24 | 144 | 1.64 | 2.38E-29 |
| hsa05322 | 21 | 93 | 1.78 | 4.19E-28 |
| hsa04217 | 16 | 149 | 1.45 | 1.96E-16 |
| hsa05203 | 14 | 182 | 1.31 | 1.86E-12 |
| hsa04110 | 7 | 120 | 1.19 | 4.57E-05 |
| hsa03010 | 7 | 130 | 1.15 | 6.36E-05 |
| hsa04919 | 6 | 119 | 1.12 | 0.0005 |
| hsa05206 | 6 | 160 | 1 | 0.0022 |
| hsa04330 | 4 | 52 | 1.31 | 0.003 |
| hsa04068 | 5 | 127 | 1.02 | 0.0064 |
| hsa05220 | 4 | 75 | 1.15 | 0.0093 |
| hsa04218 | 5 | 150 | 0.95 | 0.0113 |
| hsa05202 | 5 | 171 | 0.89 | 0.0186 |
| hsa05169 | 5 | 193 | 0.84 | 0.0294 |
| hsa05165 | 6 | 325 | 0.69 | 0.0469 |
| GO:0006325 | 60 | 713 | 1.35 | 2.52E-67 |
| GO:0051276 | 63 | 1066 | 1.19 | 2.69E-63 |
| GO:0097549 | 40 | 108 | 1.99 | 2.69E-63 |
| GO:0045814 | 38 | 103 | 1.99 | 7.13E-60 |
| GO:0040029 | 41 | 202 | 1.73 | 4.28E-56 |
| GO:0010629 | 61 | 2014 | 0.9 | 8.43E-44 |
| GO:0006333 | 34 | 193 | 1.67 | 2.14E-43 |
| GO:0045892 | 53 | 1273 | 1.04 | 2.51E-42 |
| GO:0051253 | 54 | 1422 | 1 | 1.64E-41 |
| GO:0031497 | 31 | 172 | 1.68 | 1.47E-39 |
| GO:0034728 | 30 | 176 | 1.65 | 1.44E-37 |
| GO:0006334 | 28 | 135 | 1.74 | 5.69E-37 |
| GO:0006996 | 65 | 3450 | 0.7 | 2.62E-36 |
| GO:0070828 | 24 | 68 | 1.97 | 5.29E-36 |
| GO:0071824 | 31 | 255 | 1.51 | 5.88E-35 |
| GO:0000183 | 21 | 37 | 2.18 | 1.12E-34 |
| GO:0065004 | 29 | 213 | 1.56 | 9.55E-34 |
| GO:0071103 | 32 | 328 | 1.41 | 1.84E-33 |
| GO:0031507 | 22 | 58 | 2 | 2.08E-33 |
| GO:0006338 | 28 | 206 | 1.56 | 1.78E-32 |
| GO:0045652 | 22 | 82 | 1.85 | 1.26E-30 |
| GO:0006355 | 59 | 3388 | 0.66 | 9.83E-29 |
| GO:0071840 | 69 | 5633 | 0.51 | 9.85E-29 |
| GO:0006342 | 19 | 54 | 1.97 | 4.38E-28 |
| GO:0010468 | 65 | 4813 | 0.55 | 1.39E-27 |
| GO:0006335 | 17 | 32 | 2.15 | 2.17E-27 |
| GO:0016043 | 67 | 5447 | 0.51 | 6.35E-27 |
| GO:0043044 | 20 | 91 | 1.76 | 3.26E-26 |
| GO:0060964 | 21 | 114 | 1.69 | 3.26E-26 |
| GO:0016458 | 21 | 147 | 1.58 | 3.46E-24 |
| GO:0045653 | 14 | 18 | 2.31 | 4.48E-24 |
| GO:0045637 | 24 | 260 | 1.39 | 7.53E-24 |
| GO:0034724 | 16 | 54 | 1.89 | 1.86E-22 |
| GO:0048519 | 63 | 5389 | 0.49 | 2.55E-22 |
| GO:0034080 | 15 | 43 | 1.96 | 7.39E-22 |
| GO:0016233 | 14 | 32 | 2.06 | 1.94E-21 |
| GO:0034622 | 31 | 816 | 1 | 8.53E-21 |
| GO:0032200 | 17 | 125 | 1.56 | 6.18E-19 |
| GO:0006303 | 15 | 74 | 1.73 | 7.44E-19 |
| GO:0006281 | 25 | 522 | 1.1 | 1.87E-18 |
| GO:0048523 | 57 | 4874 | 0.49 | 2.29E-18 |
| GO:0065003 | 34 | 1293 | 0.84 | 2.51E-18 |
| GO:0043933 | 36 | 1539 | 0.79 | 4.68E-18 |
| GO:0006974 | 27 | 793 | 0.95 | 1.54E-16 |
| GO:0006302 | 17 | 204 | 1.34 | 1.13E-15 |
| GO:0090304 | 37 | 2178 | 0.65 | 3.38E-14 |
| GO:0044085 | 38 | 2583 | 0.59 | 1.11E-12 |
| GO:0006352 | 15 | 214 | 1.27 | 1.22E-12 |
| GO:0044260 | 51 | 4976 | 0.43 | 1.22E-12 |
| GO:0022607 | 36 | 2359 | 0.61 | 2.90E-12 |
| GO:0044267 | 44 | 3696 | 0.5 | 3.29E-12 |
| GO:0045595 | 32 | 1874 | 0.65 | 8.61E-12 |
| GO:0043170 | 54 | 6137 | 0.37 | 5.04E-11 |
| GO:0045596 | 21 | 728 | 0.88 | 5.04E-11 |
| GO:2000026 | 32 | 2096 | 0.61 | 1.72E-10 |
| GO:0016070 | 28 | 1584 | 0.67 | 2.65E-10 |
| GO:0033554 | 29 | 1725 | 0.65 | 3.05E-10 |
| GO:0006950 | 40 | 3485 | 0.48 | 4.42E-10 |
| GO:0010467 | 31 | 2056 | 0.6 | 6.07E-10 |
| GO:0006351 | 18 | 567 | 0.92 | 6.76E-10 |
| GO:0060249 | 15 | 380 | 1.02 | 2.72E-09 |
| GO:0044271 | 26 | 1522 | 0.65 | 4.30E-09 |
| GO:0034645 | 26 | 1592 | 0.64 | 1.12E-08 |
| GO:0002682 | 25 | 1514 | 0.64 | 2.31E-08 |
| GO:0006807 | 53 | 6852 | 0.31 | 2.59E-08 |
| GO:0044403 | 19 | 865 | 0.76 | 6.58E-08 |
| GO:0016032 | 18 | 776 | 0.79 | 8.74E-08 |
| GO:0034654 | 20 | 995 | 0.73 | 9.21E-08 |
| GO:0016575 | 7 | 44 | 1.62 | 1.18E-07 |
| GO:0044238 | 53 | 7332 | 0.28 | 3.91E-07 |
| GO:0038111 | 6 | 30 | 1.72 | 5.67E-07 |
| GO:0000122 | 18 | 895 | 0.73 | 7.21E-07 |
| GO:0044237 | 53 | 7513 | 0.27 | 9.93E-07 |
| GO:0050789 | 66 | 11475 | 0.18 | 1.08E-06 |
| GO:0051241 | 20 | 1231 | 0.63 | 2.88E-06 |
| GO:0009987 | 73 | 15024 | 0.11 | 9.85E-06 |
| GO:0006614 | 7 | 96 | 1.29 | 1.48E-05 |
| GO:0016569 | 11 | 361 | 0.91 | 1.71E-05 |
| GO:0070933 | 4 | 11 | 1.98 | 2.95E-05 |
| GO:0019083 | 7 | 115 | 1.21 | 4.50E-05 |
| GO:0000184 | 7 | 119 | 1.19 | 5.56E-05 |
| GO:0006357 | 24 | 2172 | 0.47 | 9.86E-05 |
| GO:0016570 | 10 | 351 | 0.88 | 0.00011 |
| GO:0034644 | 6 | 83 | 1.28 | 0.00012 |
| GO:0044419 | 22 | 1899 | 0.49 | 0.00015 |
| GO:0006413 | 7 | 141 | 1.12 | 0.00016 |
| GO:0070932 | 4 | 20 | 1.72 | 0.00021 |
| GO:0060765 | 4 | 25 | 1.63 | 0.00045 |
| GO:0071478 | 7 | 170 | 1.04 | 0.0005 |
| GO:0050794 | 60 | 10932 | 0.16 | 0.00051 |
| GO:1901796 | 7 | 182 | 1.01 | 0.00075 |
| GO:0016579 | 8 | 281 | 0.88 | 0.0013 |
| GO:0045944 | 16 | 1253 | 0.53 | 0.0016 |
| GO:0007596 | 8 | 303 | 0.84 | 0.0022 |
| GO:0006346 | 3 | 12 | 1.82 | 0.0023 |
| GO:0033365 | 12 | 743 | 0.63 | 0.0023 |
| GO:0051716 | 42 | 6489 | 0.23 | 0.0026 |
| GO:0065008 | 31 | 4042 | 0.31 | 0.0029 |
| GO:0050896 | 48 | 8046 | 0.2 | 0.0032 |
| GO:0070914 | 3 | 14 | 1.75 | 0.0032 |
| GO:0043518 | 3 | 15 | 1.72 | 0.0038 |
| GO:0060766 | 3 | 15 | 1.72 | 0.0038 |
| GO:0072594 | 9 | 433 | 0.74 | 0.0039 |
| GO:0042060 | 9 | 439 | 0.73 | 0.0043 |
| GO:0062014 | 5 | 104 | 1.1 | 0.0053 |
| GO:0045893 | 17 | 1587 | 0.45 | 0.007 |
| GO:0007063 | 3 | 22 | 1.56 | 0.0102 |
| GO:0045815 | 4 | 62 | 1.23 | 0.0102 |
| GO:0010628 | 21 | 2337 | 0.38 | 0.0103 |
| GO:0018076 | 2 | 3 | 2.25 | 0.0125 |
| GO:0061198 | 2 | 3 | 2.25 | 0.0125 |
| GO:0070829 | 2 | 3 | 2.25 | 0.0125 |
| GO:0042592 | 17 | 1676 | 0.43 | 0.0129 |
| GO:0006310 | 6 | 219 | 0.86 | 0.0177 |
| GO:0019216 | 8 | 424 | 0.7 | 0.0186 |
| GO:0007623 | 5 | 142 | 0.97 | 0.0198 |
| GO:0016071 | 10 | 678 | 0.59 | 0.0208 |
| GO:0045935 | 18 | 1927 | 0.39 | 0.021 |
| GO:0060789 | 2 | 5 | 2.02 | 0.0241 |
| GO:0071922 | 2 | 5 | 2.02 | 0.0241 |
| GO:0010675 | 5 | 153 | 0.94 | 0.0265 |
| GO:0070647 | 12 | 1021 | 0.49 | 0.0355 |
| GO:0048511 | 6 | 271 | 0.77 | 0.0482 |
| GO:2001243 | 4 | 99 | 1.03 | 0.0482 |
| GO:0046982 | 41 | 338 | 1.51 | 1.90E-47 |
| GO:0003676 | 70 | 3947 | 0.67 | 4.33E-40 |
| GO:0003677 | 61 | 2470 | 0.81 | 9.03E-39 |
| GO:0046983 | 43 | 1037 | 1.04 | 5.11E-32 |
| GO:0005515 | 63 | 7026 | 0.37 | 2.85E-15 |
| GO:0003723 | 30 | 1649 | 0.68 | 5.25E-11 |
| GO:0019904 | 21 | 716 | 0.89 | 1.17E-10 |
| GO:0005488 | 72 | 12516 | 0.18 | 2.20E-09 |
| GO:0003690 | 20 | 1156 | 0.66 | 3.76E-06 |
| GO:0003682 | 14 | 570 | 0.81 | 1.39E-05 |
| GO:1990837 | 18 | 1068 | 0.65 | 3.11E-05 |
| GO:0008134 | 14 | 672 | 0.74 | 8.63E-05 |
| GO:0000976 | 17 | 1028 | 0.64 | 8.69E-05 |
| GO:0043565 | 19 | 1331 | 0.58 | 0.00012 |
| GO:0017136 | 4 | 15 | 1.85 | 0.0002 |
| GO:0061629 | 9 | 283 | 0.92 | 0.00035 |
| GO:0140297 | 10 | 366 | 0.86 | 0.00035 |
| GO:0033613 | 6 | 90 | 1.25 | 0.00036 |
| GO:0003735 | 7 | 159 | 1.07 | 0.00063 |
| GO:1990841 | 5 | 61 | 1.34 | 0.00099 |
| GO:0000977 | 14 | 878 | 0.62 | 0.001 |
| GO:0003712 | 11 | 571 | 0.71 | 0.002 |
| GO:0032041 | 3 | 10 | 1.9 | 0.0025 |
| GO:0031490 | 5 | 79 | 1.22 | 0.0026 |
| GO:0035257 | 6 | 155 | 1.01 | 0.0051 |
| GO:0003714 | 7 | 236 | 0.89 | 0.0056 |
| GO:0001102 | 4 | 53 | 1.3 | 0.0094 |
| GO:0002039 | 4 | 69 | 1.19 | 0.0228 |
| GO:0000979 | 3 | 26 | 1.48 | 0.0234 |
| GO:0042393 | 6 | 221 | 0.86 | 0.028 |
| GO:0051059 | 3 | 31 | 1.41 | 0.036 |
| GO:0004468 | 2 | 5 | 2.02 | 0.0361 |
| GO:0000987 | 10 | 701 | 0.58 | 0.0391 |
| GO:0097718 | 3 | 34 | 1.37 | 0.0432 |
| GO:0001103 | 3 | 35 | 1.36 | 0.0457 |
| GO:0000786 | 41 | 106 | 2.01 | 3.53E-66 |
| GO:0032993 | 42 | 195 | 1.76 | 3.14E-59 |
| GO:0000785 | 56 | 1220 | 1.08 | 1.48E-48 |
| GO:0005694 | 61 | 1712 | 0.97 | 1.48E-48 |
| GO:0000228 | 51 | 1256 | 1.03 | 2.87E-40 |
| GO:0000790 | 48 | 1048 | 1.08 | 1.48E-39 |
| GO:0043232 | 72 | 4880 | 0.59 | 2.65E-38 |
| GO:0031981 | 71 | 4733 | 0.6 | 1.81E-37 |
| GO:0000788 | 21 | 38 | 2.16 | 5.74E-35 |
| GO:0032991 | 69 | 5073 | 0.56 | 3.73E-32 |
| GO:0005654 | 53 | 3973 | 0.55 | 5.24E-19 |
| GO:0070062 | 40 | 2099 | 0.7 | 9.66E-18 |
| GO:0000784 | 15 | 102 | 1.59 | 2.66E-17 |
| GO:0042025 | 16 | 190 | 1.35 | 5.02E-15 |
| GO:0043657 | 17 | 256 | 1.24 | 1.57E-14 |
| GO:0098687 | 18 | 318 | 1.18 | 2.42E-14 |
| GO:0005615 | 41 | 3195 | 0.53 | 1.76E-12 |
| GO:0043229 | 73 | 12528 | 0.19 | 1.56E-11 |
| GO:0031982 | 41 | 3879 | 0.45 | 1.16E-09 |
| GO:0000118 | 7 | 65 | 1.45 | 6.00E-07 |
| GO:0005667 | 13 | 431 | 0.9 | 6.06E-07 |
| GO:0000792 | 7 | 76 | 1.39 | 1.56E-06 |
| GO:0022626 | 7 | 97 | 1.28 | 7.32E-06 |
| GO:0017053 | 6 | 76 | 1.32 | 3.63E-05 |
| GO:0070603 | 6 | 78 | 1.31 | 4.09E-05 |
| GO:0044391 | 7 | 178 | 1.02 | 0.00032 |
| GO:0005720 | 4 | 35 | 1.48 | 0.00071 |
| GO:0031011 | 3 | 15 | 1.72 | 0.002 |
| GO:0022625 | 4 | 56 | 1.28 | 0.0036 |
| GO:0005721 | 3 | 21 | 1.58 | 0.0046 |
| GO:0042788 | 3 | 32 | 1.39 | 0.0138 |
| GO:0022627 | 3 | 39 | 1.31 | 0.0233 |
| GO:0016580 | 2 | 11 | 1.68 | 0.045 |
| DOID:0070218 | 3 | 3 | 2.42 | 0.0044 |
| DOID:0080684 | 3 | 8 | 2 | 0.0181 |

FDR: False discovery rate; FDR<0.25 is considered statistically significant; All P<0.05.
