# Supplementary material for: H2B gene family: A prognostic biomarker and correlates with immune infiltration in glioma
Source: Front Oncol. 2022 Oct 25;12:966817. doi: 10.3389/fonc.2022.966817 (PMC9641242; doi:10.3389/fonc.2022.966817)

Supplement figure 1. Pan-cancer analysis of gene expression of H2B family


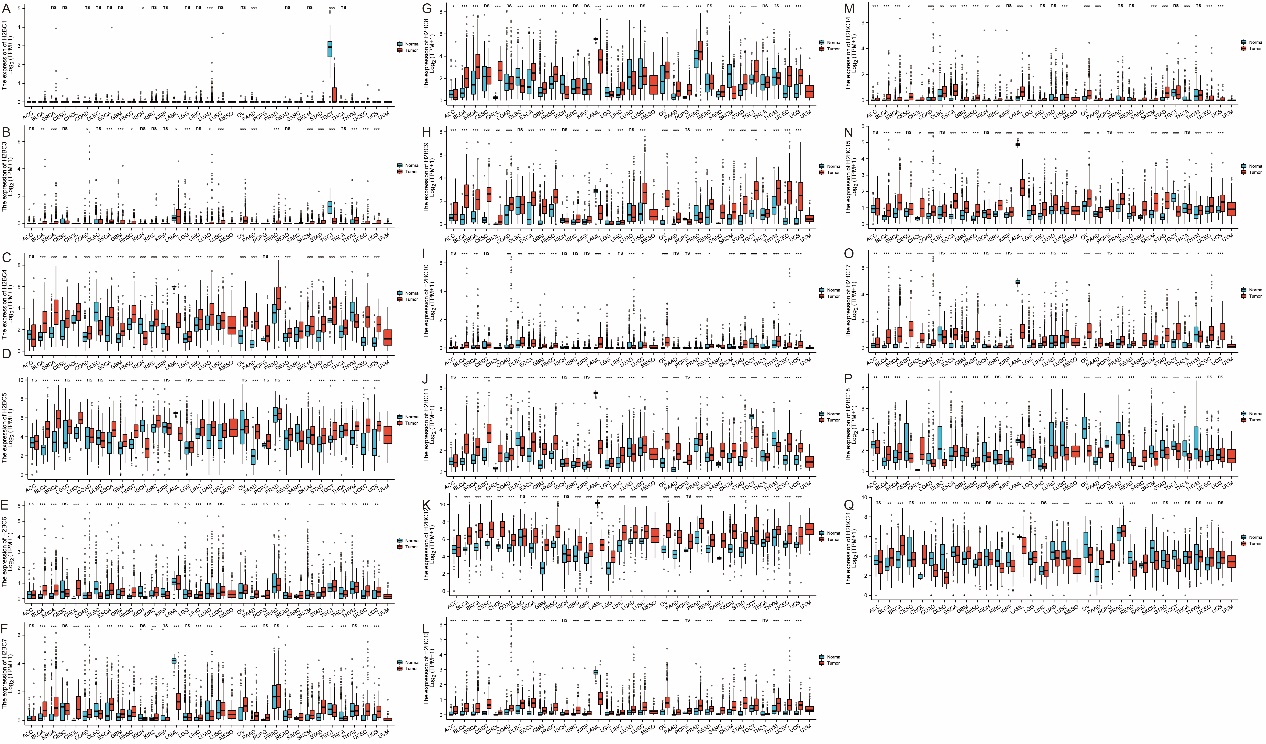


Supplement figure 2. Pan-cancer analysis of protein expression of H2B family


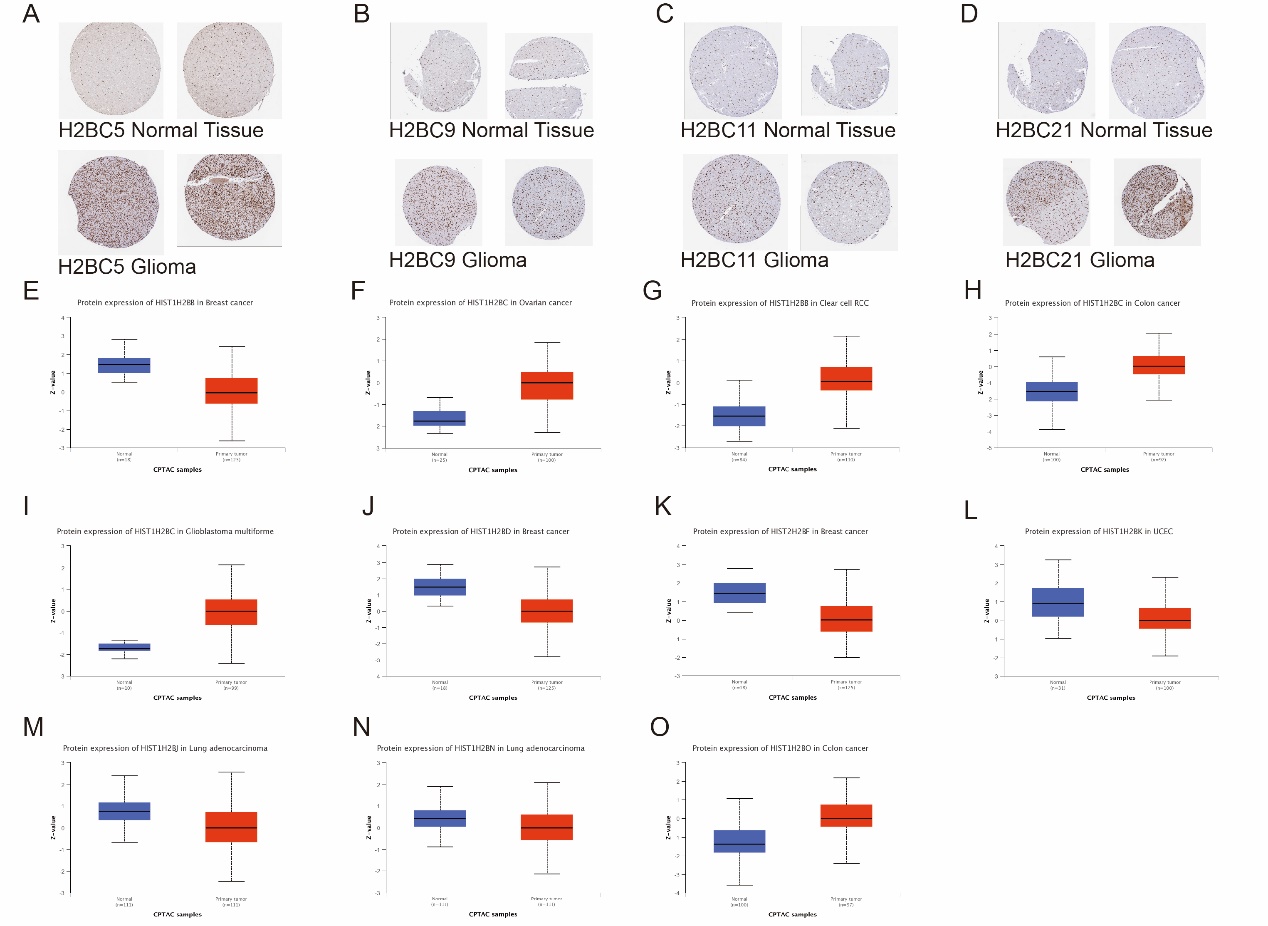


Supplement figure 3. Alteration analysis of H2B family genes


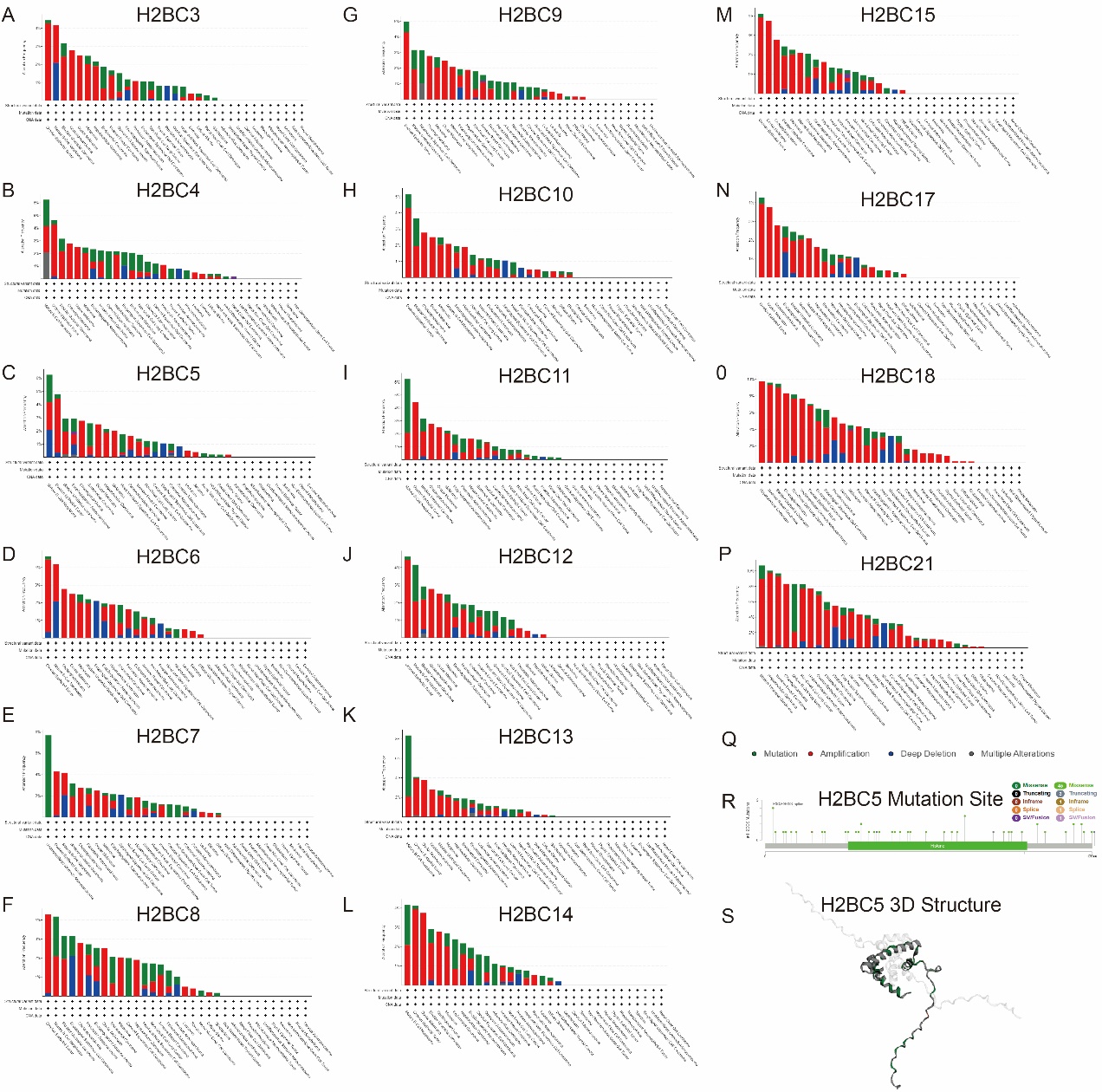


Supplement figure 4. ROC curve of H2BC5, H2BC9, H2BC11, H2BC21 and Age (from CGGA 325 and 693 cohort).


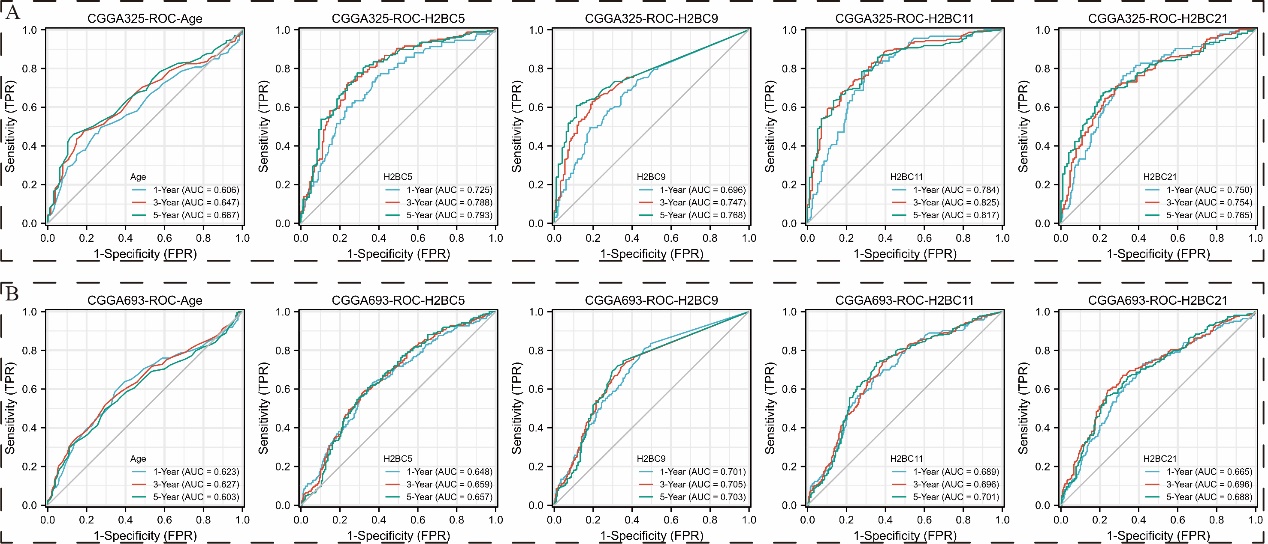


Supplement figure 5. Prognostic analysis of H2BC5, H2BC9, H2BC11 and H2BC21 in other data cohorts


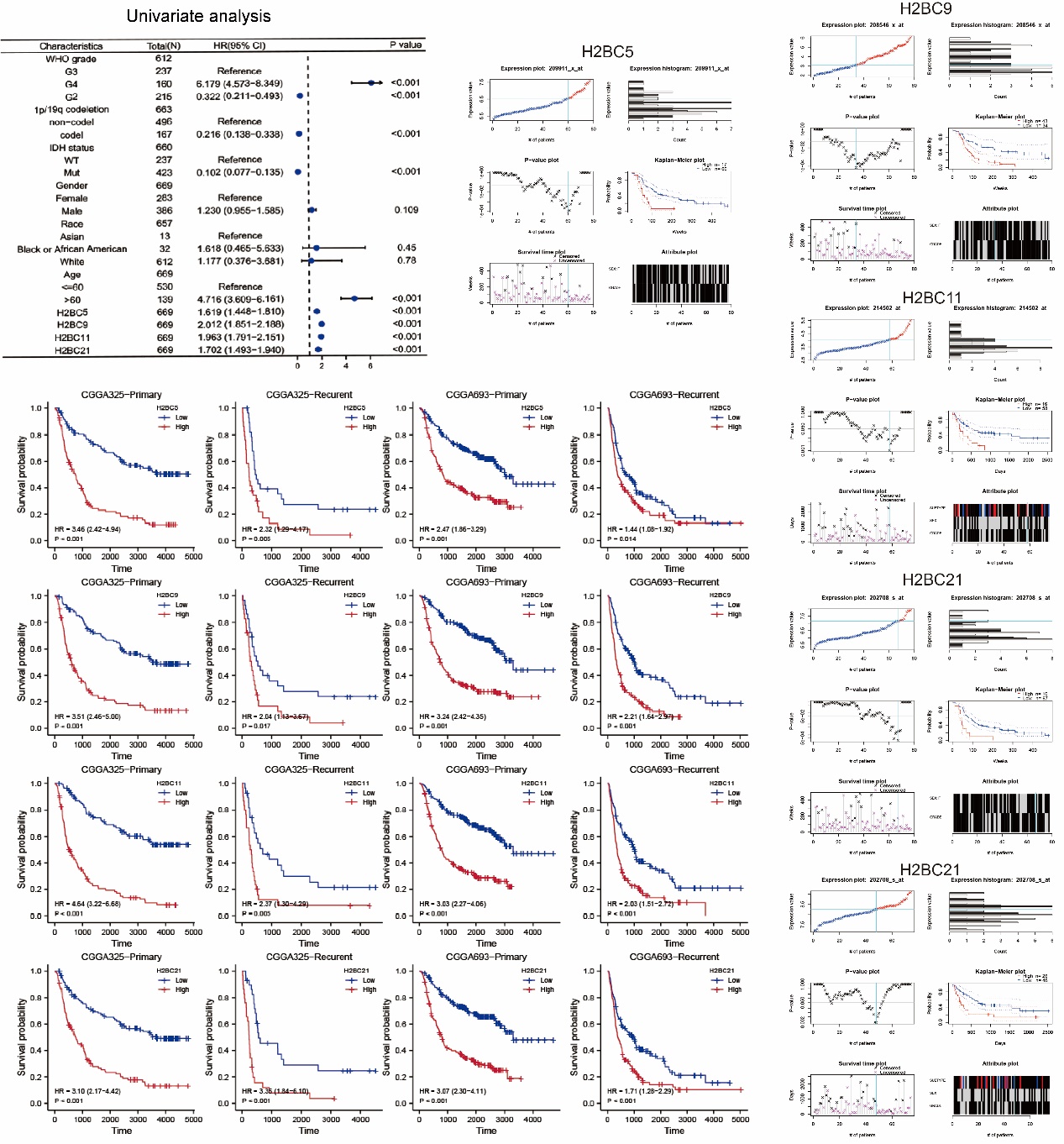


Supplement figure 6. Analysis of pan-cancer prognosis of H2B family genes.


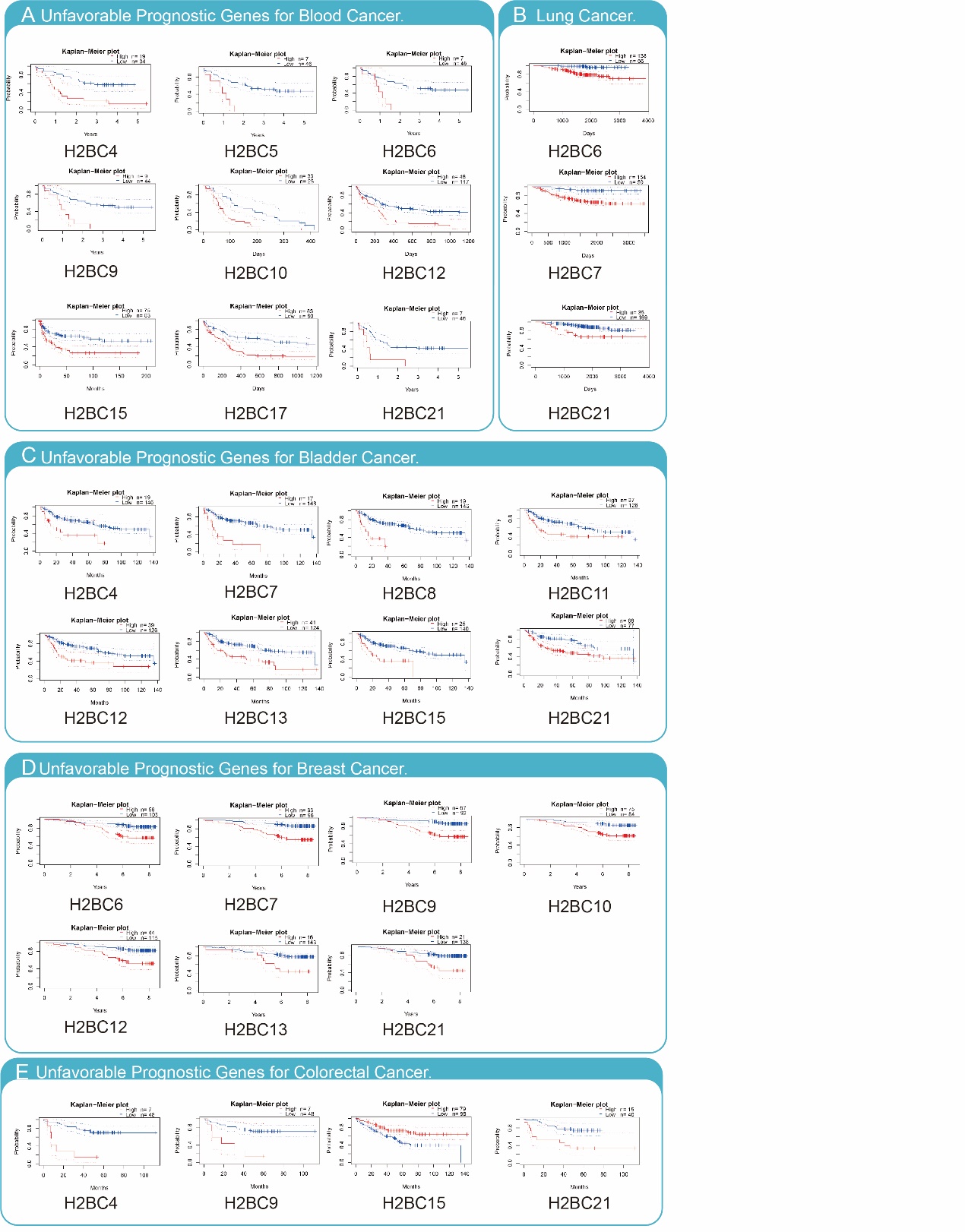


Supplement figure 7. Distribution of H2B family genes under different factors in CGGA


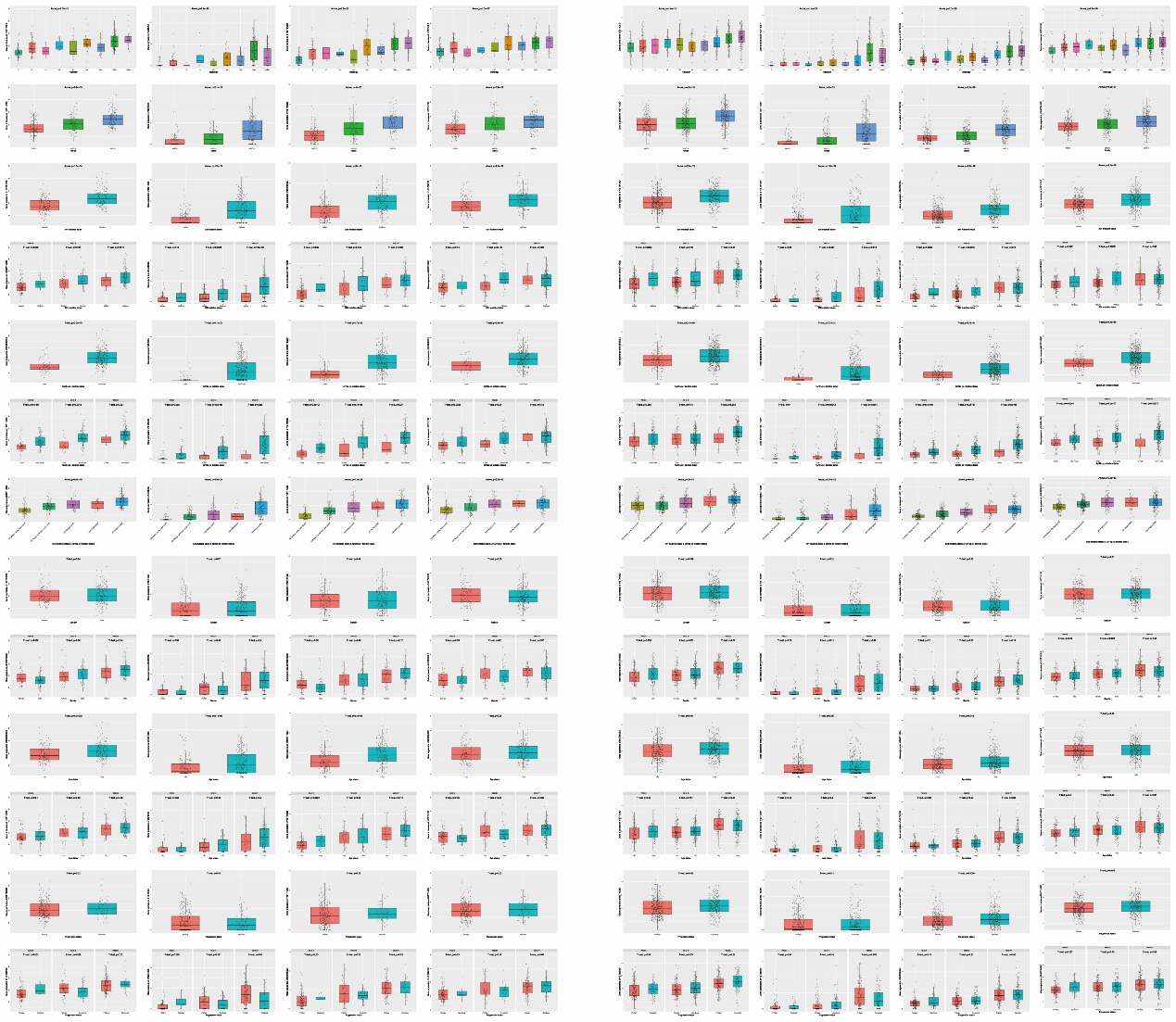


Supplement figure 8. Analysis of Immune Infiltration and Immune Subtypes of H2B Family Genes


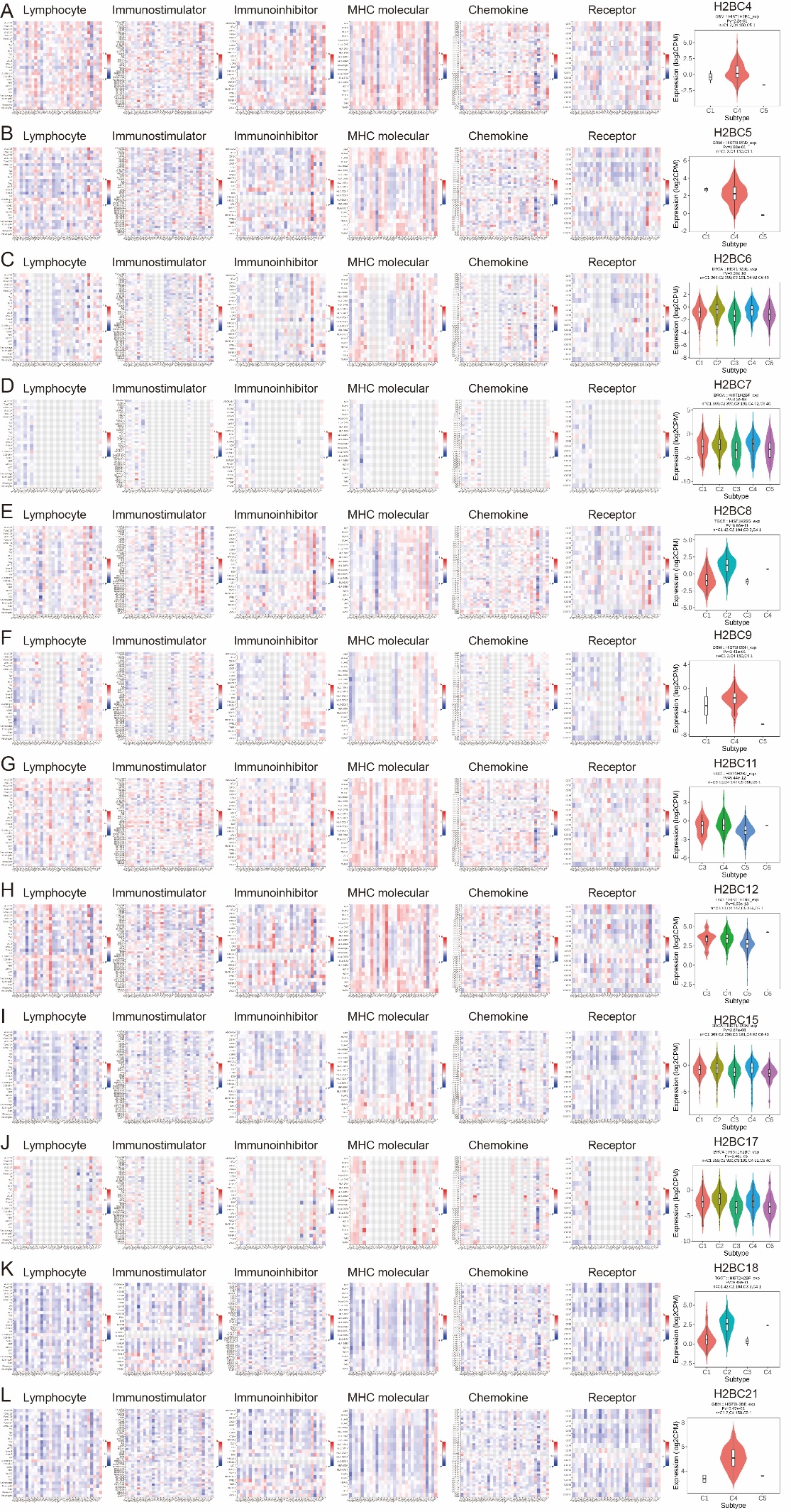


Supplement figure 9. The expression of H2BC5, H2BC9, H2BC11, H2BC21 was related to immunotherapy of Urothelial cancer.


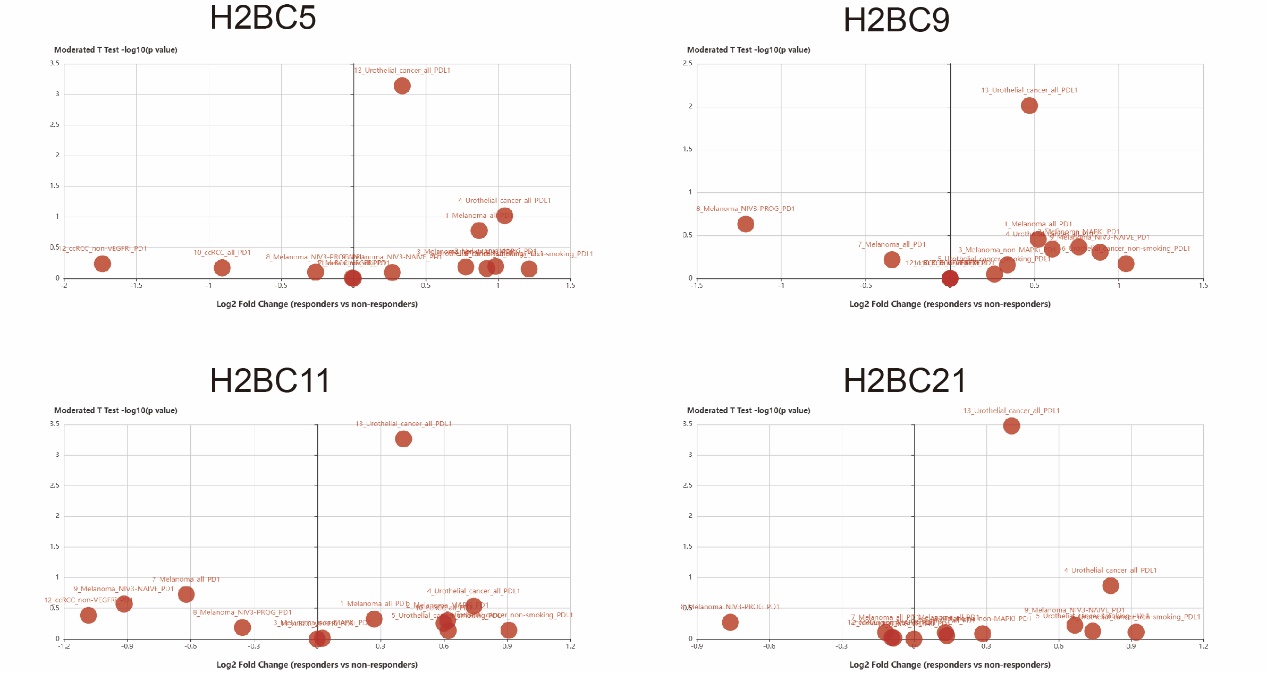


Supplement figure 10. Analysis of methylation levels of H2BC5, H2BC9, H2BC11, H2BC11 in other data sets, and correlation analysis with methyltransferase markers.


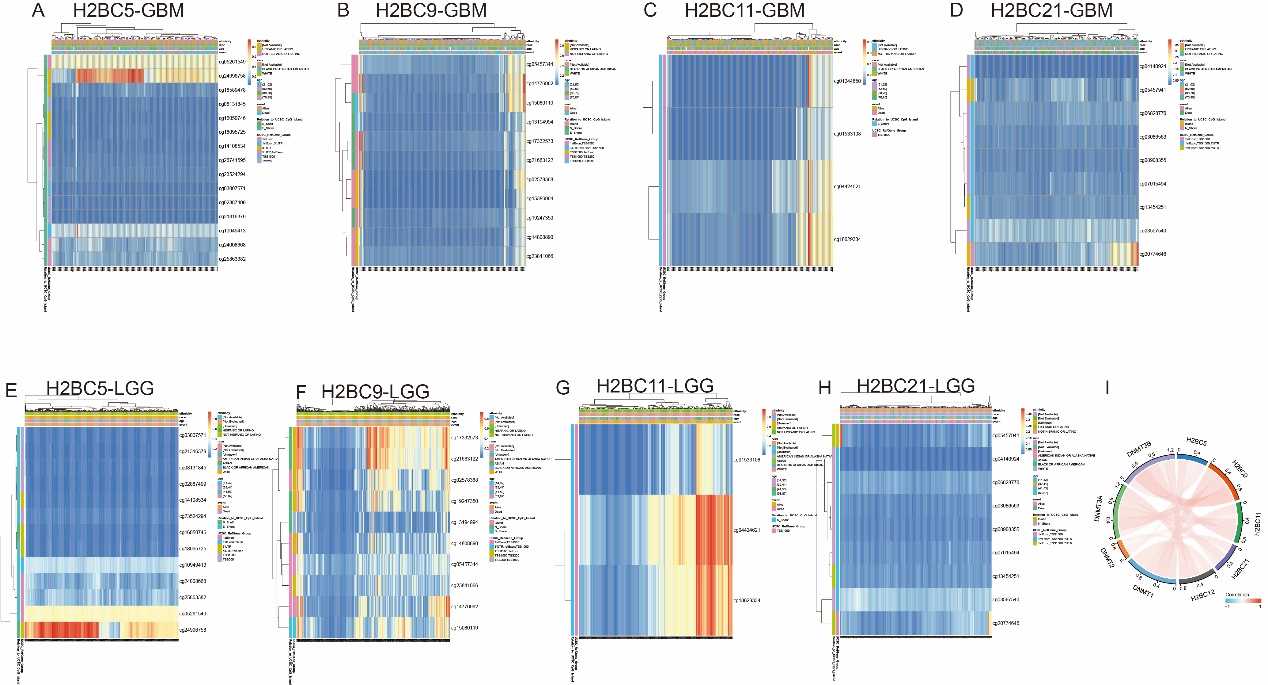

Supplement: Supplementary file 2 [file DataSheet_2.docx]
